# Potential Disruption of Flood Dynamics in the Lower Mekong River Basin Due to Upstream Flow Regulation

**DOI:** 10.1038/s41598-018-35823-4

**Published:** 2018-12-10

**Authors:** Yadu Pokhrel, Sanghoon Shin, Zihan Lin, Dai Yamazaki, Jiaguo Qi

**Affiliations:** 1 Department of Civil and Environmental Engineering, Michigan State University, East Lansing, MI 48824 USA; 2 Center for Global Change and Earth Observations, Michigan State University, East Lansing, MI 48823 USA; 3 Institute of Industrial Science, The University of Tokyo, Komaba, Tokyo Japan

## Abstract

The Mekong River Basin (MRB) is undergoing unprecedented changes due to the recent acceleration in large-scale dam construction. While the hydrology of the MRB is well understood and the effects of some of the existing dams have been studied, the potential effects of the planned dams on flood pulse dynamics over the entire Lower Mekong remains unexamined. Here, using hydrodynamic model simulations, we show that the effects of flow regulation on downstream river-floodplain dynamics are relatively predictable along the mainstream Mekong, but flow regulations could potentially disrupt the flood dynamics in the Tonle Sap River (TSR) and small distributaries in the Mekong Delta. Results suggest that TSR flow reversal could cease if the Mekong flood pulse is dampened by 50% and delayed by one-month. While flood occurrence in the vicinity of the Tonle Sap Lake and middle reach of the delta could increase due to enhanced low flow, it could decrease by up to five months in other areas due to dampened high flow, particularly during dry years. Further, areas flooded for less than five months and over six months are likely to be impacted significantly by flow regulations, but those flooded for 5–6 months could be impacted the least.

## Introduction

The Mekong River is one of the few large and complex global river systems that still remain mostly undammed^1^, but the rapid socio-economic growth, increasing regional energy demands, and geopolitical opportunities have led to a recent rise in basin-wide construction of large hydropower dams^2,3^. In the Lancang River, which drains the upper portion of the Mekong River Basin (MRB), China is building dozens of mega dams^3^; in the Lower Mekong River Basin (LMRB), some large main stem dams are being built and about a dozen main stem and over hundred tributary dams are planned^1,3,4^. The new dams and reservoirs are expected to fulfill the rapidly growing energy needs and provide other societal benefits; however, the positive benefits come with unprecedented negative social-environmental consequences^4–6^.

Fundamentally, dams alter natural flow regimes^7^ by changing the magnitude, timing, and amount of flows^8–12^, which can adversely impact downstream hydro-ecological systems, potentially causing a permanent damage to the ecological integrity of terrestrial and river-floodplain ecosystems^13,14^. In the MRB, of particular concern are the effects of flow regulation on the seasonal hydrological regime characterized by a strong unimodal flow pattern, known as the flood pulse^15^. This monsoon-driven flood pulse provides a timely supply of water and nutrient-rich sediments for flood-recession agriculture, inland fisheries, and extensive instream and wetland ecosystems, thus serving as a driving force for life and major ecosystems in the LMRB^16,17^. The flood pulse is also the primary driver of the unique flow reversal in the Tonle Sap River (TSR) that discharges water and sediments from the Mekong River into Tonle Sap Lake (TSL) during wet season and drains water from the lake into the Mekong during dry season. The seasonal river-lake inundation dynamics around TSL supports one of the world’s largest and most productive freshwater fishery^18–20^ and provides dry-season flow for critical ecosystems and agriculture in the Mekong Delta^21^. Any alterations in the duration, amplitude, timing, and rapidity of the Mekong flood pulse and the resulting changes in floodplain dynamics in the LMRB can thus severely impact a wide range of ecosystems and undermine regional food security^17^.

While the existing dams have not significantly altered the mainstream flows^22^, impacts on the tributaries have already been felt^23^. The Yali Falls dam in the Sesan River, a major tributary in the LMRB, caused an unprecedented and unpredictable flow fluctuations both during flood and dry seasons, severely damaging downstream crops, livestock, fishery, and livelihoods^23,24^. The cascade dams in the Lancang River have also affected downstream flow regimes significantly by dampening (increasing) wet (dry) season flows by 29–36% (34–155%)^25^. The reduced wet season flows could potentially offset the increased flood magnitude and variability due to climate change^26^ but the negative effects on ecosystem productivity and livelihoods caused by the variations in flood pulse could be severe and unprecedented^23^. Other studies find that the timing and magnitude of the Mekong flood pulse, which remained relatively stable historically^17^, have begun to change, likely because of upstream dam construction and climate change, making the future of TSL highly uncertain^27^. A recent study^28^ indicates that open water areas in and around the TSL have declined between 2000 and 2016, but other studies find that water infrastructure development could increase open water areas and rainfed habitats around the lake, while reducing the area of seasonally flooded habitats^27^.

Further, studies based on large-scale modeling and scenario analysis suggest that future hydropower development would dramatically alter the Mekong flow and TSL flooding^1,16,29,30^. By examining various dam development scenarios Kummu and Sarkkula^17^ suggested a drop in peak, increase in low flow, reduction in flood duration by 14 days, and decrease in flood volume and area by 7–16% around TSL. They also suggested a 17–40% increase in permanent lake area due to increased dry season flow caused by upstream dam regulation. A study by the Mekong River Commission (MRC^31^), however, suggests a reduction (increase) in flood depth during wet (dry) season, which could potentially reduce the total flooded areas by 400–900 km^2^, forest-covered flood areas by 22–100 km^2^, grasslands by 50–150 km^2^, and rice fields by 300–630 km^2^. Using hydrological model simulations and climate change scenarios, Arias *et al*.^27^ found that climate change could increase water levels during wet seasons in TSL, but water infrastructure development would reduce water levels. Similar findings have been reported in other studies that combined different scenarios of climate change and infrastructure development^16,22,30,32,33^.

As synthesized in Pokhrel *et al*.^3^, the growing body of literature has provided a good understanding of the surface hydrology of the LMRB and TSL^16,34,35^. However, the potential effects of flow regulation by dams and the likely hydrologic regime shifts due to climate change on flood dynamics in the entire LMRB still remain a subject of intense debate^3^. Further, while great strides have been made in understanding the changes in flooding around TSL, the precise role of seasonal flood pulse in modulating the terrestrial water storage (TWS; see Supplementary Information Section 1) variations and river-lake inundation dynamics remains unexamined, especially by considering the TSR flow reversal effects. Such role of surface water in modulating the basin hydrology has been extensively studied using models and data from the Gravity Recovery and Climate Experiment (GRACE) satellites for other tropical river basins such as the Amazon^36,37^ and Congo^38,39^, but rarely reported for the Mekong to the authors’ best knowledge. The goal of this study is to address these gaps by seeking answer to the following science questions: (1) What is the role of seasonal flood pulse and TSR flow reversal in modulating the TWS variations in the MRB? (2) What are the potential impacts of changes in flood pulse due to upstream flow regulation on river-lake flood inundation dynamics in the LMRB?

We answer the first question by analyzing the results from a combination of hydrological (HiGW-MAT^40^) and hydrodynamic (CaMa-Flood^41,42^) models, and the TWS data from GRACE satellites (see Methods). The second question is answered by examining different scenarios of flow regulation designed by altering the magnitude and timing of flood pulse near Stung Treng (see Methods), a location in the vicinity of the proposed site for the massive Sambor dam with 18 km barrier, which, if built, is feared to severely fragment the river dolphin population and block fish migration^43,44^. Our approach is novel in that we use the altered flow patterns over scenarios of flow regulation by the existing and planned dams because the existing dams have caused little hydrologic alterations in mainstream flows^22^, and the number, construction time, and size of dams to be built remains highly uncertain. We note that our flow alteration patterns are not designed to perfectly capture the actual flow regulation by any specific future dam, but provide a framework for sensitivity analysis under varying degrees of flow regulation by a single of multiple dams in the upstream of the location where we alter the flows. Further, while future dams could be operated for different purposes, our analysis is based on flow regulation patterns of hydropower and flood control dams whereby a reduced flood peak is accompanied by an increased low flow. Thus, our approach enables a mechanistic understanding of the changes in downstream flooding under different levels of potential flow regulation by any upstream dams. Further, while previous studies have mostly focused on the TSL, we examine the TWS variations across the entire MRB and flood dynamics in the LMRB including the Mekong Delta, providing a relatively complete picture of potential dam impacts.

## Results

Results are presented in three sections. The first section provides model evaluation and the remaining two address each of the research questions.

### Model Evaluation

**Figure 1:**
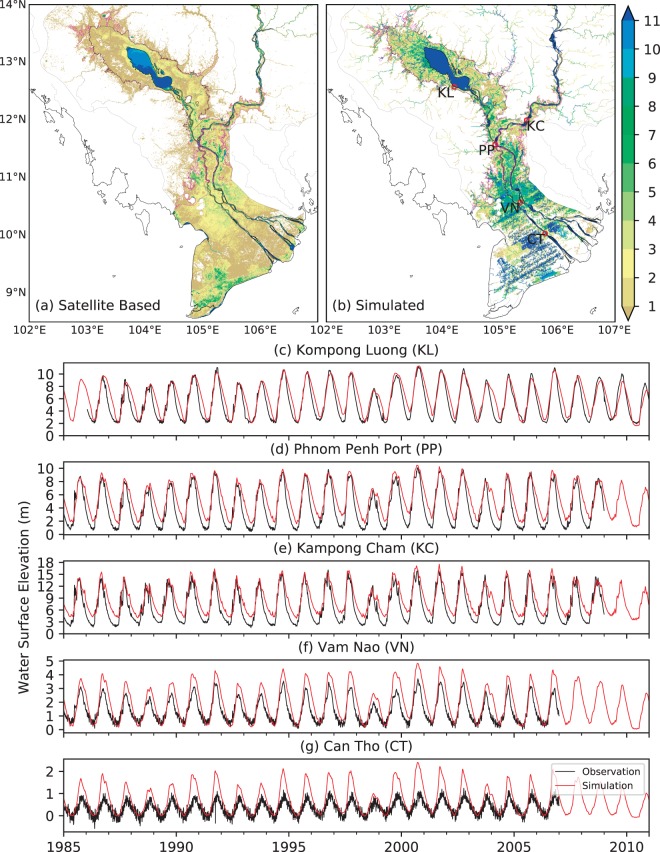
Monthly flood occurrence and daily water surface elevation. (**a**,**b**) Comparison of simulated flood occurrence (number of months) with satellite-based flood occurrence^49^. The region enclosed by magenta lines shows the areas of major flood around TSL and Lower Mekong within Cambodia (source: https://data.humdata.org/), and the thick black outline marks the flooded areas around TSL used in previous studies^27^. (**c**–**g**) Comparison of simulated (CaMa-Flood) and observed (obtained from MRC) water surface elevation at five stations indicated by red circles in (**b**). Observations are shown only for the period available.

Both the HiGW-MAT and CaMa-Flood models have been extensively validated both globally^10,11,40,41,45–47^ and over the MRB^42,48^. Thus, here we keep the evaluations brief with a focus on variables related to flood estimation, specifically flood occurrence (i.e., number of flooded months per year) and water surface elevation. Modeled flood occurrence, derived from the flood depth downscaled to 500 m grids (see Methods and Supplementary Fig. S1), is compared with the satellite-based 30 m global data (upscaled to 500 m) of historical water occurrence^49^ (Fig. 1a,b). A good agreement can be observed in terms of the broad patterns of flooded areas, but discrepancies are evident in flood occurrence itself. Notable differences can be seen around the northwest portion of the TSL, where numerous previous studies^17,22,50^ as well as our results suggest a permanent water occurrence (i.e., 12-month flood occurrence) but the satellite data indicate nonexistence of such permanent water. This possible underestimation of permanent water occurrence in the satellite data results from underestimated water occurrence during April-July, likely due to the presence of relatively shallow and turbid water.

Further, the model simulates high flood occurrence around the main body of the lake, along river channels, and the flat floodplains in the Mekong Delta, expected but not seen in the satellite data. This could be a possible model overestimation caused by the uncertainties in topographic and climate data, or an underestimation in the satellite product, which represents “open to sky” water presence, potentially underestimating water occurrence under vegetated areas such as in the gallery forests and flood-recession agriculture around the lake and in the delta region^51^. Moreover, while the model provides a continuous simulation of monthly flood occurrence, only limitedly available cloud-free images are used in the satellite product^49^. Note that the stripes in the Mekong Delta region (Fig. 1b) result from small but inherent errors in the digital elevation model (DEM) in low-lying areas^52^. For TSL region, the model clearly captures the areas of major flood (magenta lines in Fig. 1a,b). Further, comparison of simulated flooded areas with the estimates from a previous study^27^ for the major flooded regions around TSL (black outline in Fig. 1a) suggests that the model well captures the total flooded areas both during dry and wet seasons (Supplementary Table S1).

Since the satellite data could contain uncertainties, we evaluate the modeled water surface elevation—a primary determinant of flood extent, depth, and occurrence—with the ground-based observation to add further confidence to our flood simulations (Fig. 1c–g). Evidently, both the seasonal magnitude and temporal variability of water elevation are well captured by the model, especially at the Kompong Luong (KL) in the TSL and Phnom Penh Port (PP). Simulated water levels are not as accurate in the lower portion of the delta (e.g., Can Tho; Fig. 1g), which could be due to the uncertainties in DEM, river width, and channel bathymetry represented in our river bifurcation scheme^42^. At Can Tho, discrepancies could also be attributed to tide effects, not considered in the current model. Given the scale of the model domain, uncertainties in data, and the difficulty in accurately representing channel bifurcation, we consider these results to be reasonable for this study.

### Role of River-Floodplain Storage on TWS Dynamics and Historical Variability

**Figure 2:**
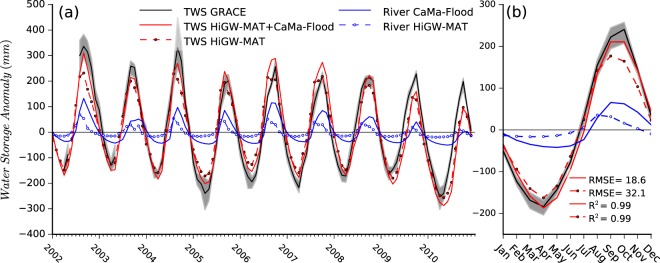
Role of river-floodplain storage on TWS dynamics over the MRB. (**a**) Black line shows the mean of TWS anomaly from spherical harmonics- and mascon-based GRACE products with the range between different products indicated by grey shading. For modeled TWS anomalies, four sets of result are shown: combined total TWS from HiGW-MAT and CaMa-Flood (solid red), total TWS only from HiGW-MAT (dashed red), river-floodwater storage from CaMa-Flood (solid blue), and river water storage from HiGW-MAT (dashed blue) which implicitly lumps flood water storage. (**b**) The seasonal cycle. Results are averaged for the entire MRB.

Next, we examine the role of river-floodplain water storage in modulating the TWS dynamics in the MRB using TWS variations simulated by the models and from GRACE satellites (see Methods). By comparing the TWS solely from HiGW-MAT, the combined TWS from HiGW-MAT and CaMa-Flood, and TWS from GRACE, we find that river-floodplain storage plays a critical role in modulating the total TWS variations, and hence the hydrology of the MRB (Fig. 2). First, the variations in river-floodplain storage from CaMa-Flood (solid blue line) exhibit substantially larger seasonal amplitude than the river storage (dashed blue line) in HiGW-MAT that does not consider TSR flow reversal and lacks an explicit representation of floodwater storage. While certain inter-annual variations are obvious, the differences can be clearly discerned from the seasonal cycle (Fig. 2b). Second, a one-month delay in the peak can be seen in river-floodplain storage in CaMa-Flood as compared to the river storage in HiGW-MAT, which expectedly results from larger floodplain storage in CaMa-Flood during wet season—partly due to TSR flow reversal—and a subsequent release in the dry season.

Third, as a consequence of larger seasonal swing and delayed peak in CaMa-Flood, the combined TWS from HiGW-MAT and CaMa-Flood (see Methods) provides a better agreement with GRACE compared to the TWS from HiGW-MAT alone. The better agreement is reflected not only graphically, but also statistically; while the already-high R^2^ (0.99) does not change, the root mean squared error (RMSE) reduces from ~32 to ~18 mm (Fig. 2b). Because GRACE provides the vertically integrated total TWS, not its components^37^, river-floodplain storage from CaMa-Flood could not be separately validated, but an independent evaluations of water level (Fig. 1c–g) and river discharge (Supplementary Figs S2, S3) suggest that CaMa-Flood well simulates the overall hydrodynamics. Fourth, comparison of the seasonal amplitude in the combined TWS (Fig. 2b; solid red line) and the river-floodplain storage from CaMa-Flood (Fig. 2b; solid blue line) suggests that river-floodplain storage explains ~27% of the seasonal amplitude of total TWS variations averaged over the entire MRB as opposed to only ~13% by river storage in HiGW-MAT (Fig. 2b; dashed blue line); for the LMRB, while CaMa-Flood river-floodplain storage contributes to ~49% of total TWS, HiGW-MAT river storage only accounts for ~12% (Supplementary Fig. S4). These findings imply that the potential alterations in the Mekong flood pulse and TSR flow reversal will affect not only the dynamics of flood patterns but also the overall basin hydrology because changes in surface water storage can alter other components of the basin water balance.

**Figure 3:**
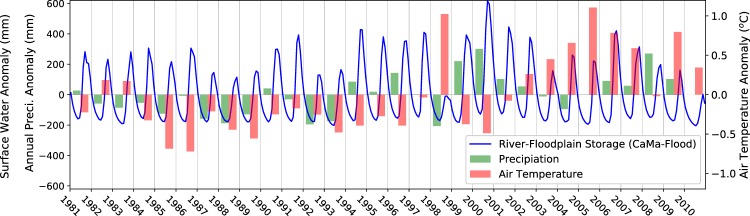
Relationship between river-floodplain storage from CaMa-Flood (monthly) and climate variability (annual mean precipitation and temperature) over the Lower Mekong domain shown in Fig. 1a,b. Precipitation and temperature data are same as those used as input to HiGW-MAT model^40^.

Figure 3 shows the historical variations in river-floodplain storage from CaMa-Flood over the Lower Mekong domain under varying climate conditions (i.e., annual precipitation and temperature). Evidently, annual storage variations are largely dictated by the variabilities in inter-annual precipitation (correlation of 0.67) and temperature (correlation of −0.31). High precipitation, often combined with low basin-wide temperatures, lead to wet years and vice versa; however, no significant trend in river-floodplain storage (Mann-Kendall test, p = 0.958, α = 0.05) is found over the 30-year period. The lowest and highest storages clearly stand out in years 1998 and 2000, which are among the driest and wettest years, respectively in the past few decades^20,53^; flood occurrence in these years is discussed further in the next section. Note that these results do not account for the effects of existing dams, but such effects are relatively small compared to the flow volume in the main stem of the Mekong (Supplementary Fig. S5).

### Potential Effects of Flow Regulation on Flood Dynamics in the LMRB

**Figure 4:**
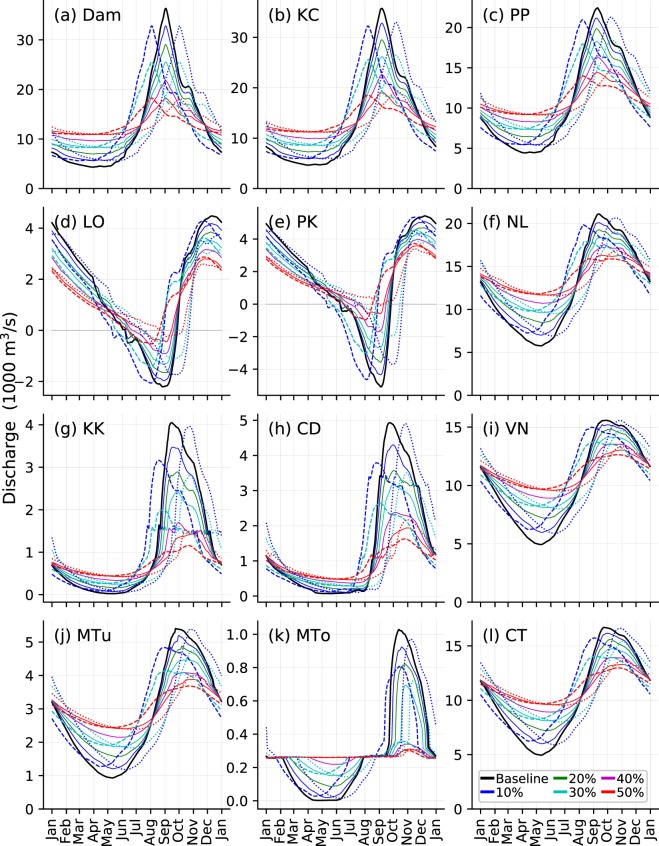
Daily river discharge at locations shown in Fig. 5b. The thick black line shows the baseline flow (1981–2010 average); other colors represent different scenarios of change in peak flow magnitude (solid lines). Dashed and dotted lines represent the scenarios of early and delayed peak timing, respectively, by one month for different degree of peak flow alteration represented by the color coding. Results of altered timing are shown only for 10, 30, and 50% peak flow reduction scenarios. Station names are: KC (Kampong Cham), PP (Phnom Penh Port), LO (Lake Outlet), PK (Prek Kdam); NL (Neak Luong), KK (Koh Khel), CD (Chau Doc), VN (Vam Nao), MTu (My Thuan), MTo (My Tho), and CT (Can Tho); latitudes and longitudes are provided in Supplementary Table S6.

**Figure 5:**
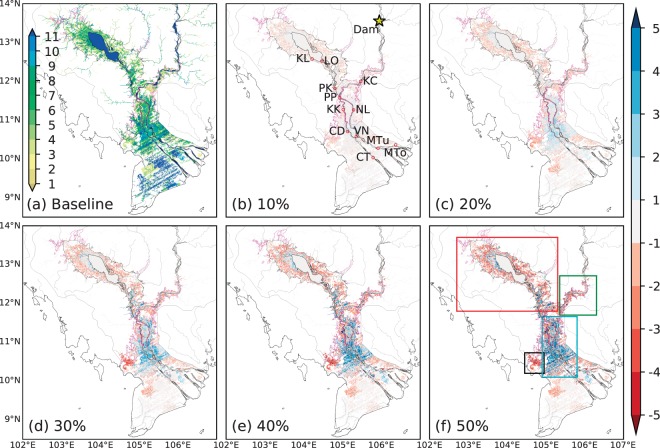
Flood occurrence and the effects of flow regulation on it. (**a**) Same as in Fig. 1b but based on the baseline simulation with 1981–2010 mean runoff. (**b**–**f**) Change in flood occurrence (number of months) for change in peak flow by different degree as indicated with reference to the baseline flood occurrence (**a**). The star in (**b**) shows the Stung Treng station where flow is altered. Rectangles in (**f**) show regions in the LMRB discussed in the text. For magenta and black lines, see description in Fig. 1 caption.

Figure 4 presents the potential effects of upstream flow regulation (altered magnitude and timing of peak near Stung Treng, marked by a star in Fig. 5b; see Methods) on downstream flow dynamics in the mainstream Mekong, TSR, and some distributaries in the delta region (locations shown in Fig. 5b). Note that only the magnitude and timing of peak are altered, and mass balance is preserved in all flow regulation scenarios (see Methods). Results in Fig. 4 represent a surrogate of an average year, defined as the mean for 1981–2010 period; typical dry and wet years are discussed next. Up to the PP station, highly similar altered flow patterns are observed to that at the dam location (Fig. 4a–c) but interesting features emerge in the downstream of PP and in the TSR. Most notably, upstream flow alterations are found to severely impact the magnitude, timing, and direction of discharge into TSL (LO and PK stations; Fig. 4d,e), which could potentially disrupt the natural flood dynamics in the TSR and cause a regime shift in TSL water balance. Results suggest that, for different flow regulation scenarios, the peak of flow from the TSL to the Mekong would reduce by 7–37% and 7–34% (Supplementary Table S2a) and that of the reversed flow from the Mekong into TSL by 11–80% and 15–88% (Table S2b) at LO and PK stations, respectively. Together, the changes in the peaks of the bi-directional flow in the TSR would dampen the seasonal amplitude (i.e., maximum-minimum) of the hydrograph at LO and PK stations by 8–51% and 10–60% (Table S2c), respectively, under different upstream flow regulation scenarios. These changes in flood dynamics could significantly alter the onset, duration, and amount of flow reversal in the TSR. We find that the onset could be delayed by 1–38 days and 1–40 days (Table S2d), respectively, at LO and PK stations, with a reduction in the total duration of reversed flow by 2–51 days and 2–55 days (Table S2e). As a result, the total volume of water entering the TSL due to flow reversal could reduce from 12,202 (23,646) million m^3^ by 14–87% (15–92%) at the LO (PK) station for different flow alteration scenarios (Table S2f).

In the Mekong Delta region, flow regulations cause relatively predictable changes in river flow dynamics along the mainstream channels, but flood dynamics becomes highly unpredictable in the distributaries and bifurcated channels. In the main channels (e.g., NL, VN, and CT stations; Fig. 4f,i,l), the alterations in the magnitude of seasonal amplitude (i.e., dampened peak and enhanced low flow) show a similar pattern to the upstream flow alterations with a more pronounced increase in low flow than the reduction in flood peak. This interesting phenomenon in the downstream of the confluence of the TSR results partly from the weakened flow reversal in the TSR, meaning that because less water flows into the TSR during wet season, the mainstream flow in the downstream of PP station is not significantly affected by TSR flow reversal. Consequently, in dry season, most of the enhanced low flow from the upper main stem is directly discharged to the downstream with considerably less contribution from TSR flow. That is, while the TSL plays a role of a detention reservoir by dampening flood peak and enhancing low flow in the downstream, such role becomes less effective when the mainstream flow is regulated by upstream reservoirs reducing the wet-season flow into the lake.

At the KK and CD stations (Fig. 4g,h) which are on the Bassac River, a distributary of the Mekong originating near PP, flood peak is substantially reduced due to the dampened upstream flood peak; however, the increase in low flow is relatively small but distributed over a longer period than at the mainstream stations. Understanding these potential changes in high and low flows is crucial because the Bassac River is a critical transportation corridor between Cambodia and Vietnam. Moreover, in these downstream locations likely increase in water levels due to sea level rise and tides could interfere with the changes brought by upstream dam regulation, causing unpredictable flow and water level patterns. Further downstream, highly unpredictable flows are observed at MTo (near My Tho in Vietnam) station (Fig. 4k), because of unpredictable changes in channel bifurcation dynamics; here, flood seasonality potentially ceases under high upstream flow regulation and delayed peak scenarios (Table S2a,b). Note that some of these changes could have resulted from the uncertainties in channel bifurcation simulations in our model in the low-lying areas as discussed earlier.

Figure 4 also includes the results from simulations with one month early and delayed peak at the dam location. Overall, the changed timing of peak results in correspondingly shifted hydrograph in the immediate downstream of dam location; however, the altered timing is found to affect also the magnitude of peak flow in the TSR and delta region. At LO and PK stations (Fig. 4d,e), the compounded effects of reduced peak and altered timing cause an even larger impact on the timing, duration, and amount of reversed flow than that caused only by reduced peak (also see Table S2d–f). Notably, results suggest that TSR flow reversal at PK station almost ceases if the flood peak reduced by 50% arrives with a one month delay relative to the baseline flow (Fig. 4e, Table S2b). In the delta region (e.g., KK and CD; Fig. 4g,h), delayed (early) timing is found to enhance (dampen) the flood peak magnitude, suggesting that there is an optimum timing for flood patterns to be maintained at the base level; any changes in timing can causes a significant increase or decrease in the flood peak magnitude. The changes in water surface elevation (Fig. S6) are found to follow similar patters to the changes in discharge.

Figure 5 presents the changes in flood occurrence within the same spatial domain shown in Fig. 1. Evidently, downstream impacts vary among different scenarios of flow regulation. First, the changes are relatively small for 10% and 20% peak reduction. Second, increased flood occurrence can be seen at the vicinity of TSL and along the main river channels because of increased water retention during dry season. Away from the lake and in the flooded agricultural areas, flood occurrence decreases significantly (by up to 5 months or more) because of large decline in flood water entering the TSL from the Mekong during wet season. Overall, flooded areas in the TSL region (thick black line within the red rectangle in Fig. 5f) averaged for the high flood season (August-October) decrease by 413 km^2^ (4.6%), 774 (8.6%), 1122 (12.5%), 1602 (17.8%), and 2075 (23.1%) for 10, 20, 30, 40, and 50% scenarios, respectively. Similarly, flooded areas during dry season (April-June) increase by 93 km^2^ (2.9%), 311 (9.7%), 580 (18.1%), 862 (26.9%), and 1144 (35.7%). Results suggest that the flooded areas in the floodplains upstream of PP (green rectangle in Fig. 5f) also decrease significantly under all flow alteration scenarios. Third, no significant change in water occurrence can be observed within the main body of TSL, suggesting the presence of permanent water under all flow alteration scenarios, which could partly be attributed to a simple treatment of lake bed elevations owing to the inherent limitations in DEM data that provide only the water surface elevation over water bodies and the lack of spatially-explicit bathymetry data. Thus, these results should be interpreted with caution.

Fourth, in the Mekong Delta region, a large increase in flood occurrence can be seen in the middle reach (post-flooding agricultural areas; cyan rectangle in Fig. 5f) for >30% flow regulation. Again, this results from a relatively small impact on the mainstream flow during flood season as less water enters the TSL and an increase in low flow because of dam release (Fig. 4f,i,l). Farther from the mainstream channels in the lower portion of the delta, flood occurrence mostly decreases because lowered water levels (Fig. S6j,k) in the mainstream channels prevent frequent overtopping to the floodplains. Fifth, no changes in flood occurrence can be seen in mainstream Mekong and Bassac Rivers as well as other distributaries near the river mouth. In these regions, the magnitude and timing of water levels are simulated differently under different scenarios, but the water occurrence remains unchanged because of permanent water occurrence in the model. Finally, our results indicate that while the areas flooded for ~6 months are least impacted by flow regulation during average years, the areas flooded for ~1 (~12) months could decrease (increase) significantly under all flow alteration scenarios and both in the TSL region as well as the entire Lower Mekong domain (Fig. S7). This is a direct consequence of the reduced flood peak and increased low flow under all flow regulation scenarios. In terms of the impacts of changed flood peak timing, the effects tend to become smaller with increased duration of flood occurrence; in general, regions that are flooded over nine months are minimally impacted both in the Lower Mekong and TSL regions (Fig. S7). Similar patterns were reported in a previous study^22^ in that the reductions in peak and increases in low flows are amplified for higher degrees of flow regulation. Similarities are found also in terms of the least impacted flood occurrence (in general, 40–60%, which is similar to ~6 months in a year). These evaluations are summarized in Supplementary Table S3, but it is noted that the results are not directly comparable because of the differences in simulation settings (see footnotes in Table S3), which results in considerably different baseline simulations (Two-sample K-S test, p = 0.11, α = 0.05).

**Figure 6:**
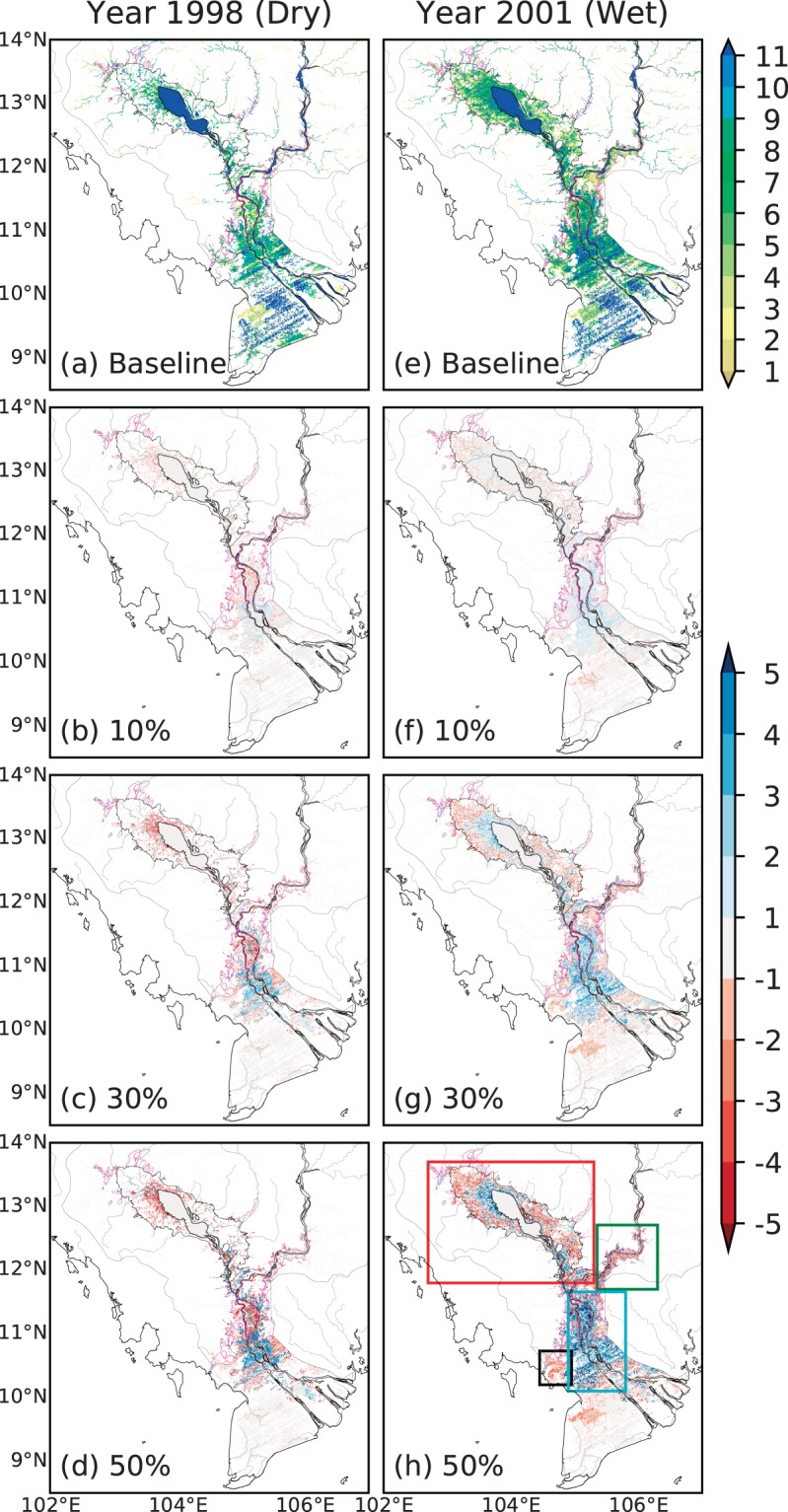
Same as in Fig. 5 but for dry (1998) and wet (2000) years. For the altered flow scenarios, results for only 10, 30, and 50% alterations are shown. Rectangles in (h) show regions in the LMRB discussed in the text. For magenta and black lines, see description in Fig. 1 caption.

Because the effects of flow regulation on downstream flood patterns can vary significantly during dry and wet years, we examine the results for 1998 and 2000 (Figs 6 and S8), which represent the historical dry and wet years, respectively^17,27^ (Figs 1 and 3). Although the broad spatial patterns of changes in flood occurrence during dry and wet years appear similar to those during the average year (Fig. 5), magnitudes vary, and some interesting features emerge. Substantially smaller (larger) flooded areas and occurrence can be seen during dry (wet) years (Fig. 6a,e) compared to that in an average year (Fig. 5a). Specifically, during the high flood season (August–October), 51.3% (36.9%) more (less) areas are flooded in wet (dry) years compared to the average year. Similarly, during the dry season (April–June), 17.1% (1.4%) more (less) areas are flooded in wet (dry) years. Further, for the 10% flow alteration scenario, marked differences are not found in downstream flood occurrence between dry, normal, and wet years. However, varying patterns of change in flood occurrence become readily discernable between dry and wet years for the 30%, and even more so for the 50% scenario. In the wet year, substantial areas in the western vicinity of the TSL experience an increase in flood occurrence by up to 6 months for 50% scenario, but the same region experiences a notable decline in flood occurrence during the dry year (Fig. 6d,h). As in normal year (Fig. 5f), a marked reduction in flood occurrence is seen in the outer extents of the major flooded areas around TSL (shown by magenta line) in both dry and wet years (Fig. 6d,h). No change in flood occurrence that can be seen northwest of the TSL flooded areas in 50% scenario for 1998 (Fig. 6d) is in fact due to no flood occurrence in all scenarios including the baseline (Fig. 6a**)**.

The effects of flow regulation on the seasonal flood dynamics (similar to Fig. 4) during dry and wet years are presented in the Supplementary Material (Figs S9, S10), along with the changes in different flood characteristics (Tables S4, S5) for all flow alteration scenarios. Evidently, the peak flood magnitude decreases and the low flow increases, resulting in significantly dampened flood pulse amplitude in all Lower Mekong stations (Tables S4, S5). Notably, the TSR flow reversal tends to completely cease under 50% peak reduction scenario during dry year (Fig. S9d,e, Table S4d,e), which is likely also during the wet year if the flow under 50% peak reduction scenario is delayed by one month (Fig. S10d,e, Table S5d,e). The onset of TSR flow reversal tends to shift in the same direction and by a comparable duration to the duration of altered peak timing (i.e., one month) for 10% scenario but the effects are varying for 30% and 50% scenarios (Tables S2, S4, S5). The other flood characteristics (e.g., duration and volume of reversed flow) are also affected to a considerably varying extent due to the alteration of timing under different scenarios of peak flow reduction (Tables S2, S4, S5).

In the downstream of PP station (cyan rectangle in Fig. 6h), as in the average year, substantial increase in flood occurrence is seen during the wet year (especially for >30% peak alteration scenarios) which is primarily due to a longer retention of water in these flat areas caused by increased low flow (Fig. 4g,h) and higher dry-season water levels (Fig. S6g,h). On the contrary, a significant reduction in flood occurrence can be seen in the upper portion of this region in the dry year because the relatively small increase in baseflow does not lead to a sustained flood water during the low flow season. In the areas upstream of PP station (green rectangle in Fig. 6h), flooding is primarily caused by water overtopping river banks during wet season, thus the reduced flood peak causes a marked decline in flood extents and occurrence under all scenarios during both dry and wet years. Finally, within the region near the Long Xuyen Quadrangle (black rectangle in Fig. 6h), a large decline in flood occurrence seen during a normal year (Fig. 5b–f) is not evident during the wet year (Fig. 6f–h) because of significantly larger flows in the wet year even in the 50% regulation scenario (see Figs 4 and S10); in the dry year no change can be seen because this region is rarely flooded (Fig. 6a).

## Conclusions

We find that river-floodplain water storage plays a crucial role in modulating the hydrology of the MRB, and that the potential upstream flow regulations could disrupt the natural flood dynamics in the TSL region and Mekong Delta. Results indicate that the river-floodplain water explains ~26% of the total storage dynamics in the MRB and ~49% in the LMRB, suggesting that the potential flow alterations can largely modify the natural regime of the Lower Mekong hydrology. It is found that the reduction in the peak of flood pulse by more than 20% near Stung Treng gauging station could cause a significant alteration in the water balance of the TSL, potentially ceasing the flow reversal in the TSR and disrupting the lake inundation dynamics, if the flood peak at the same location is dampened by 50% and delayed by one-month. During average and wet years, flood occurrence could increase at the outer fringe of the permanent water in the TSL and post-flooding agricultural regions in the middle reach of the Mekong Delta; however, during dry years flood occurrence could reduce by up to 5 months or more around the outer edge of the flooded areas in the TSL region, in the flood-recession agricultural region at the vicinity of the Mekong upstream of Phnom Penh, and downstream portion of the Mekong delta. Further, while areas flooded for less than five months and over six months are likely to be impacted significantly by flow regulations, areas flooded for 5–6 months could be impacted the least. These results provide new insights about how the downstream flood dynamics could change under different levels of upstream flow regulation by proposed dams, which have important implications for sustainable hydropower development to ensure food security and ecological integrity in the Mekong region. Interpretations should be made with caution because our results represent possible future scenarios of downstream impacts due to varying degrees of upstream flow regulation but not the actual impacts of any specific dam.

## Materials and Methods

### Model Description

Two models are used; the first is the global hydrological model HiGW-MAT^40^, which is based on the global land surface model (LSM) MATSIRO^54^ coupled with the river routing model TRIP^55^. HiGW-MAT simulates both the natural water cycle and human activities such as irrigation, flow regulation, and groundwater pumping, but the human impact schemes are turned off because the objective here is to examine the effects of potential flow regulation by future dams, not limited to the existing ones; note that the existing dams have caused little impact on the Mekong flow^22^. MATSIRO simulates key vegetation, surface hydrological, soil moisture, and groundwater processes on a full physical basis. A complete description can be found in Takata *et al*.^54^, with further details on its recent version and integration of TRIP in Pokhrel *et al*.^10,40,56^. The MATSIRO-TRIP framework has been extensively used and validated globally^10,11,40,45,46,57,58^.

The other model is the global hydrodynamic model CaMa-Flood^41,42^, which computes river hydrodynamics (i.e., river discharge, flow velocity, water level, and inundated area) by solving shallow water equation of open channel flow, explicitly accounting for backwater effects using the local inertial approximation^47^. We use CaMa-Flood version-3.6 with regional settings at 10 km resolution for the MRB^42^, which includes the capability for downscaling output to 500 m grids; version-3.6 accounts for channel bifurcation, a critically important process to realistically simulate river-floodplain dynamics in the Mekong Delta. In CaMa-Flood, water level and inundated areas are diagnosed from water storage in each unit catchment; river discharge from each unit catchment is calculated using the shallow water equation; water storage at each unit catchment is updated by a mass conservation equation considering discharge input from the upstream unit catchment(s), discharge output to the downstream unit catchment, and local runoff input from HiGW-MAT. The 10 km river network map is generated by upscaling the 3 arc-second (90 m) HydroSHEDS flow direction map^59^ and digital elevation model from SRTM3 DEM^42^. Manning’s roughness coefficient for rivers and floodplains is set basin wide at 0.03 and 0.10, respectively, following Yamazaki *et al*.^41,42,60^; sensitivity of the coefficient to model results is discussed in Yamazaki *et al*.^41^. All other model parameters including river width are identical to those in Yamazaki *et al*.^42^. CaMa-Flood has been widely used for regional- to global-scale flood analysis^41,42,47^. Further details can be found in our previous publications^41,42,47^.

### Simulation Settings

First, the HiGW-MAT model is used to simulate runoff and all TWS components (i.e., soil moisture, snow, river storage, and groundwater) for 1979–2010 period using identical settings, parameters, and forcing data as in Pokhrel *et al*.^40^; the first two years are discarded as spinup; results for 1981–2010 are analyzed. Since HiGW-MAT is a global model, results for the MRB (90–110°E, 5–35°N) are extracted from global simulations at 1° grids. Runoff is used to drive the CaMa-Flood model and the storage components are used for TWS analysis. Note that the HiGW-MAT is used for a single simulation without considering flow alterations.

Then, using a similar approach as in our previous studies^42,48^, daily runoff from HiGW-MAT is used in CaMa-Flood to simulate river-floodplain hydrodynamics at 10 km over the MRB. All simulation settings and model parameters are identical to those in Yamazaki *et al*.^42^. The 10 km resolution flood depth is then downscaled to 500 m grids using SRTM3 high-resolution DEM following Yamazaki *et al*.^42^. A series of simulations are conducted for: (1) 1981–2010 period using continuous HiGW-MAT runoff and with no flow alterations; (2) an average year, using the climatological mean daily runoff for 1981–2010 period; (3) a historical dry year (1998); and (4) a historical wet year (2000). For (2), (3), and (4), first a baseline simulation is conducted without flow alterations. Then, simulations with varying degrees of dampened flood peak (i.e., by 10, 20, 30, 40, and 50%) and early and delayed arrival of the peak by one month (see details below) are conducted. These scenarios are designed to capture the reduction in magnitude and a delay in timing of the flood peak caused typically by hydropower and flood-control dams. The early peak represents a possible scenario of changed timing under climate change. While these scenarios may not capture the actual flow regulations by future dams, they represent the plausible scenarios of the cumulative effects of upstream dams, similar to those observed in other large river basins such as the Colorado^56^.

Following Felfelani *et al*.^46^, simulated TWS components from HiGW-MAT are vertically integrated to calculate TWS anomalies averaged over the MRB for 2002–2010, an overlapping period between GRACE and simulations. TWS thus derived includes river storage simulated by a simple routing model TRIP^55^ but excludes floodwater storage. Then, another set of TWS time series is derived by replacing the river storage in HiGW-MAT-based TWS by the river-floodplain storage from CaMa-Flood, and without altering the other TWS components. The two sets of TWS are then compared with the TWS from GRACE to examine the role of river-floodplain storage in modulating TWS variations. Note that the river storage in HiGW-MAT and river-floodplain storage in CaMa-Flood are simulated using the same runoff from HiGW-MAT, thus the mass balance in TWS computations is preserved. The component contribution of river-floodplain water to the total TWS is calculated as the ratio of seasonal amplitude of river-floodplain storage to the seasonal amplitude in the simulated total TWS^37^.

### Data

Historical observations of river discharge and water level are obtained from the MRC. For the analysis of TWS variations, we use both the Spherical Harmonics and mascon^61^ based GRACE products. Spatially averaged time series over the entire MRB is generated following a similar approach as in our previous study^46^. Additional details on TWS data processing are provided in the Supplementary Information Section 1.

### Altered Flood Pulse Magnitude and Timing

To generate the altered flood pulse patterns as a surrogate of flow regulations by future dams, we change the timing and magnitude of flood peak near Stung Treng gauging station (13.53°N, 105.95°E) in the Mekong river, immediate downstream of the confluence of the 3S river systems, a location near the proposed site for the massive Sambor dam^43^. This approach enables us to mechanistically examine the changes in flood magnitude, timing, duration, and extent under different levels of dam regulations or altered flow patterns due to climate change. Although the majority of the proposed large dams are likely to be used for hydropower generation, no information is available on how these dams will be operated. However, as most dams do, the new dams will alter the magnitude and timing of river flow by attenuating the peak and increasing low flow. Thus, to capture these altered flow patterns, we generate a proxy of dam release using a release equation proposed by Hanasaki *et al*.^9^ and used in our recent study^62^ to simulate the effects of existing dams, which can be written as:

1 $${Q}_{i,DAM}={Q}_{mean}\times (M+(1-M)\frac{{Q}_{i,NAT}}{{Q}_{mean}})$$

where, *Q*_*i*,*DAM*_ is the altered flow, *Q*_*i*,*NAT*_ is the simulated natural flow at the dam location, and *Q*_*mean*_ is the mean annual natural flow for each operational year. *M* is a calibration parameter that determines the release; e.g., when *M* is unity, the equation represents a constant release throughout the year, and when *M* is zero, release is equal to the natural flow, representing no reservoir effect. Here, we calibrate *M* to attenuate peak by 10, 20, 30, 40, and 50% from the baseline (i.e., average year) flow. Because *Q*_*mean*_ is different for each year, *M* values are differently calibrated among years, i.e., average (1981–2010 mean), dry (1998), and wet (2000), to maintain the same degree of peak flow attenuation. Once *Q*_*mean*_ and *M* are determined, *Q*_*i*,*DAM*_ is generated using equation (1) that produces enhanced low-flow to compensate for peak flow reduction, preserving water balance. For the scenarios with altered timing of peak, *Q*_*i*,*DAM*_ is derived by shifting the peak of the hydrograph one month earlier or later. Note that the scenarios of altered timing are analyzed only for 10, 30, and 50% peak flow attenuation scenarios. The baseline and altered flow for average, dry, and wet years are shown in Figs 4a, S9a and S10a, respectively.

## Electronic supplementary material

Supplementary Information
