# Supplementary material for: Potential Disruption of Flood Dynamics in the Lower Mekong River Basin Due to Upstream Flow Regulation
Source: Sci Rep. 2018 Dec 10;8:17767. doi: 10.1038/s41598-018-35823-4 (PMC6288158; doi:10.1038/s41598-018-35823-4)
Supplement: Supplementary file 1 — Supplementary Information [file 41598_2018_35823_MOESM1_ESM.pdf]

Supplementary Information

Potential Disruption of Flood Dynamics in the Lower Mekong River Basin Due to Upstream Flow Regulation

Yadu Pokhrel[1], Sanghoon Shin[1], Zihan Lin[2], Dai Yamazaki[3], and Jiaguo Qi[2]

[1]Department of Civil and Environmental Engineering, Michigan State University, East Lansing, MI 48824.

[2]Center for Global Change and Earth Observations, Michigan State University, East Lansing, MI 48824.

[3]Institute of Industrial Science, The University of Tokyo, Komaba, Tokyo, Japan.

1. Terrestrial Water Storage (TWS) and its Estimation

TWS is composed of water stored over and underneath the land surface; thus, it is estimated by vertically integrating snow water, canopy water, river and floodplain water, soil water, and groundwater storages over a given spatial domain, typically a river basin. Mathematically, this can be expressed as[1]:

TWS = Surface water + Subsurface water
Surface water = FW + RW + SW + CW (1)
Subsurface water = VW + GW
Where, FW = water on the floodplains
RW = water in the river channels
SW = snow water
CW = water stored in canopy surfaces
VW = soil water in the vadose zone (unsaturated store)
GW = groundwater (below the water table, saturated store)

The TWS derived from the measurements made by the Gravity Recovery and Climate Experiment (GRACE) satellite mission[2] provides the vertically-integrated TWS and thus includes all components listed in Equation (1). In hydrological models such as HiGW-MAT[3], however, each of the components is typically simulated on an individual basis. Thus, vertically integrated TWS for comparison with GRACE-based TWS is estimated by adding all components using Equation (1).

As briefly described in the "Materials and Methods" section in the main text, we use two different GRACE products. The first is a set of three Spherical Harmonics (SH) products[4] available from three different processing centers: (i) the Center for Space Research (CSR), (ii) the Jet Propulsion Laboratory (JPL), and (iii) the German Research Center for Geoscience (GFZ). These products are available for download at: https://grace.jpl.nasa.gov/data/get-data/monthly-mass-grids-land/. The second is the mascon product[5] available from two different processing centers: JPL (https://grace.jpl.nasa.gov/data/get-data/jpl_global_mascons/) and CSR (http://www2.csr.utexas.edu/grace/RL05_mascons.html). Both the SH and mascon products are readily available in terms of equivalent water height, thus no additional processing (i.e., filtering and smoothing) is applied in this study. We generate the spatially-averaged TWS time series for the entire MRB for each of the products as described below.

Because GRACE measures the TWS variations over large regions, the GRACE data and model results are typically compared as basin averages[1,6,7] over river basins having an area larger than the GRACE footprint[8] of ~200,000 km^2. In this study, we estimate the basin-averaged TWS from both GRACE and HiGW-MAT model by taking an area-weighted average following the approach used in our recent study[6]:

$$H(x,t) = \frac{\sum_{i=1}^{n} S_i(x,t)}{A}, \quad S_i(x) = \begin{cases} 1 \times s \times a_i & \textit{inside the basin} \\ 0 & \textit{outside the basin} \end{cases}$$

where s is the LSM or GRACE estimate, a_i is the cell area, S_i is the weighted estimate for each cell inside the basin, n is the number of cells in a basin, A is the total area of the basin, and $H(x,t)$ represents the estimate of water storage for basin at time t.

As briefly described in the "Methods" section in the main text, two sets of basin-averaged TWS are derived from the model results. In the first set, the flood water (FW) component in Equation (1) doesn't exist because river-floodplain storage is lumped in the river water (RW) component of the TRIP[9] routing model used in HiGW-MAT[3]. In the second set, FW based on the explicit simulation by the CaMa-Flood[10] model is included. For uniformity, CaMa-Flood results at 10km grids are first upscaled to the grid resolution of GRACE data and HiGW-MAT model (i.e., 1°×1°). The basin boundary for the entire Mekong is used as shown in Fig. S2.

2. Simulated Flood Depth and Evaluation of River Discharge

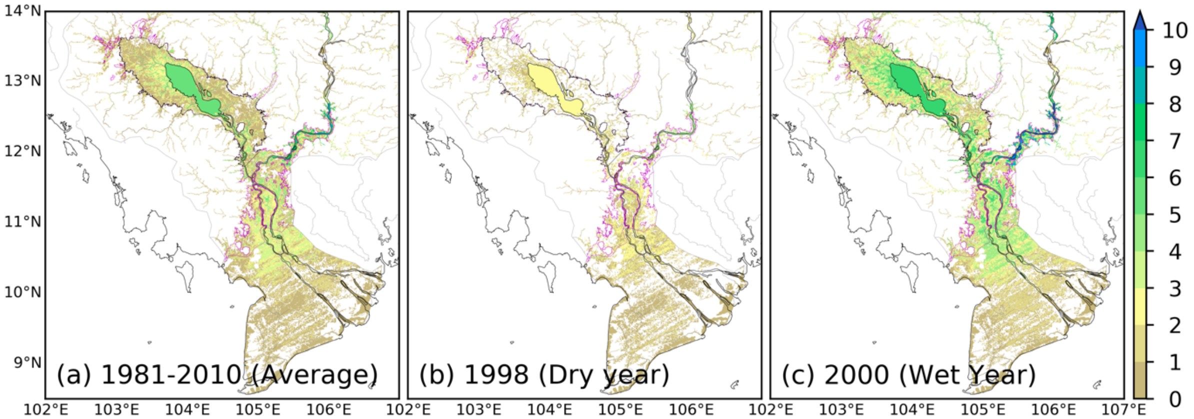

Figure S1. Simulated annual mean flood depth downscaled to 500m spatial resolution using high resolution SRTM[11] topography data for (a) average year (mean of 1981-2010), (b) dry year (1998), and wet year (2000). The region enclosed by magenta lines shows the areas of major flood around Tonle Sap Lake (TSL) and Lower Mekong within Cambodia (source: https://data.humdata.org/), and the thick black outline marks the flooded areas around TSL used in previous studies[12]. The domain displayed is same as that shown in Figure 1 in the main text.

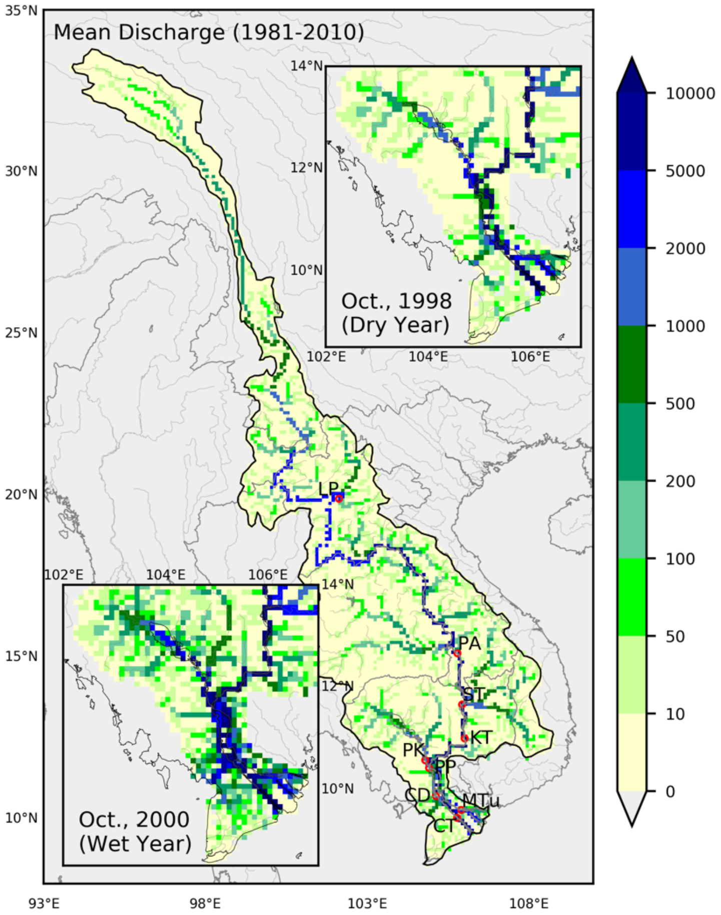

Figure S2. Long-term (1981-2010) mean river discharge (m^3/s) simulated by CaMa-Flood at 10km spatial resolution over the entire Mekong River Basin (MRB). The upper right and lower left insets show river discharge for the Lower Mekong during peak flow season in 1998 (dry year) and 2000 (wet year), respectively. Red circles show the locations for river discharge validation presented in Figure S3; station names are: LP (Luang Prabang), PA (Pakse), ST (Stung Treng), KT (Kratie), PP (Phnom Penh Port), PK (Prek Kdam), CD (Chau Doc), CT (Can Tho), and MTu (My Thuan).

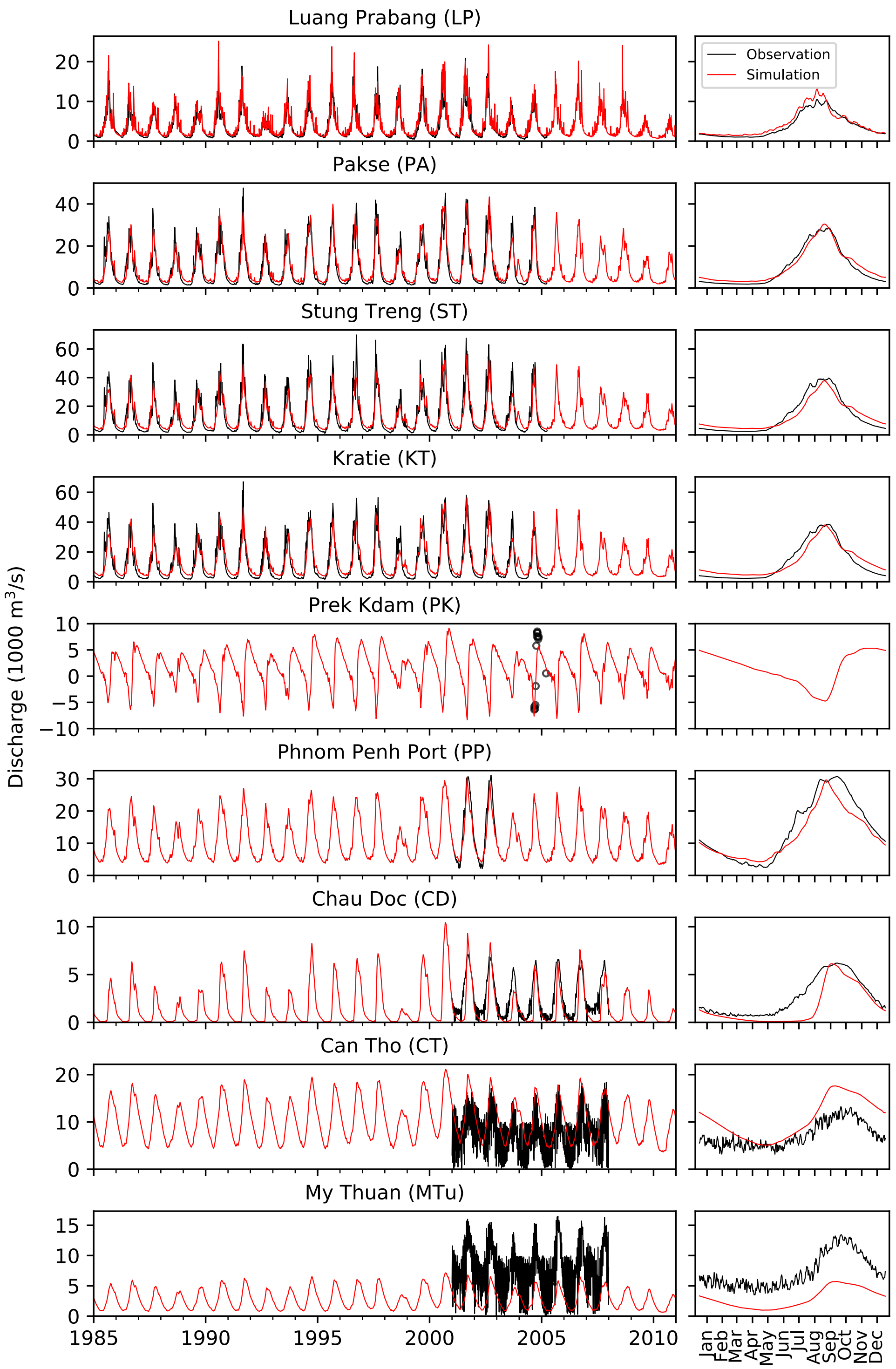

Figure S3. Evaluation of simulated river discharge with observations obtained from the Mekong River Commission (MRC) at locations indicated by red circles in Figure S2. While the simulated results are shown for the period of 1985-2010, observed data are shown for the period available. The right panels show the daily climatological mean over the period for which observations were available. For PK station, limited data were available only for year 2004.

3. Contribution of Surface Water to Total TWS in the Lower Mekong

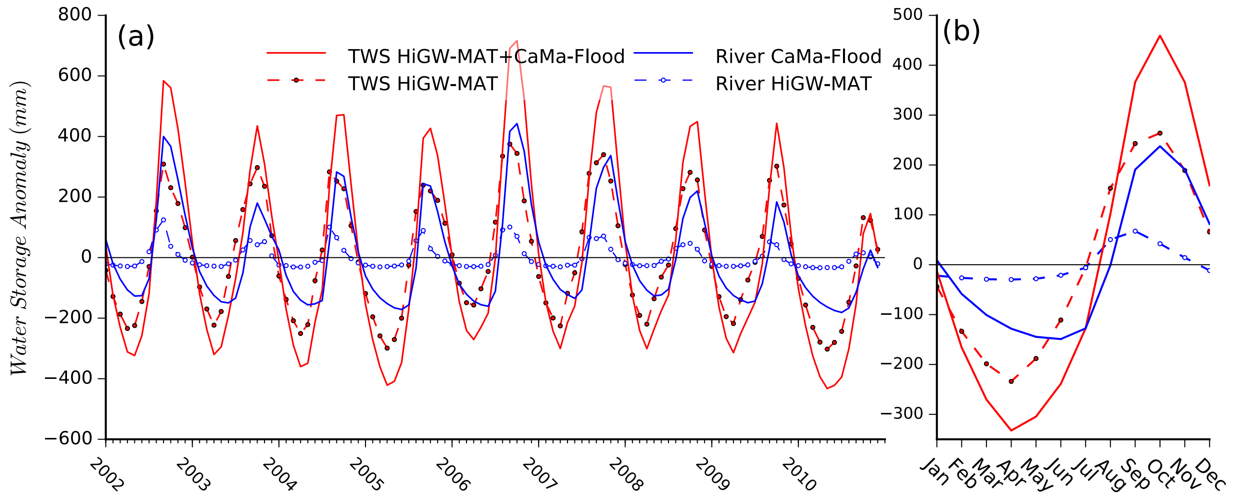

Figure S4. Same as in Fig. 2 in the main text but only for the Lower Mekong region shown in Fig. 1. Note that GRACE data are not included here because of the reduced reliability of the data when averaged over small regions.

4. River Discharge from Simulations with and Without Existing Dams

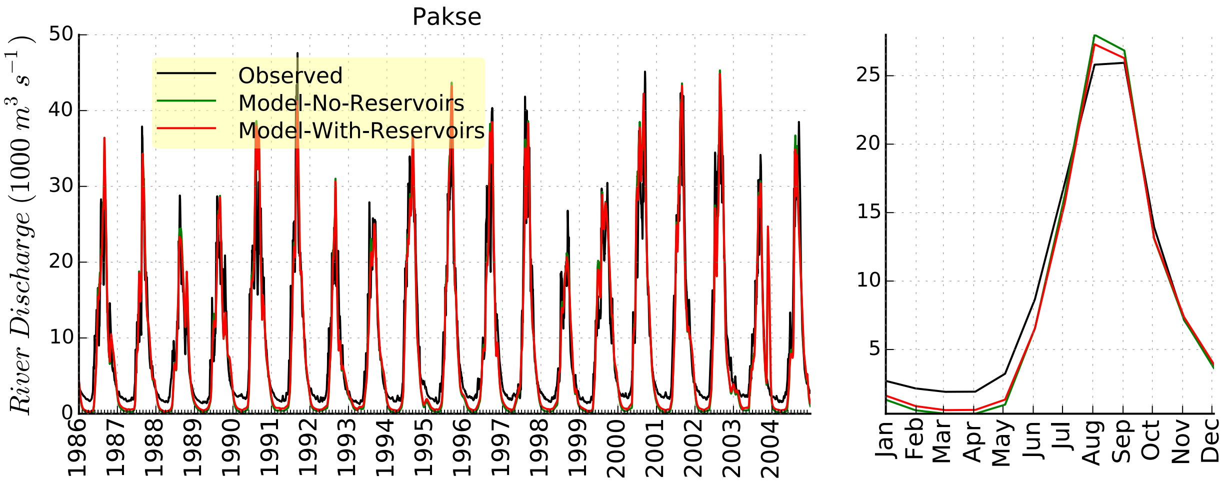

Figure S5. Daily river discharge at Pakse (PA) station (location shown in Figure S2) simulated by HiGW-MAT model with and without considering the existing dams. Observed data from MRC are also shown. The right panel shows the monthly seasonal cycle. Model results are taken from Pokhrel et al.[3].

5. Effects of Upstream Flow Regulation on Downstream Water Levels

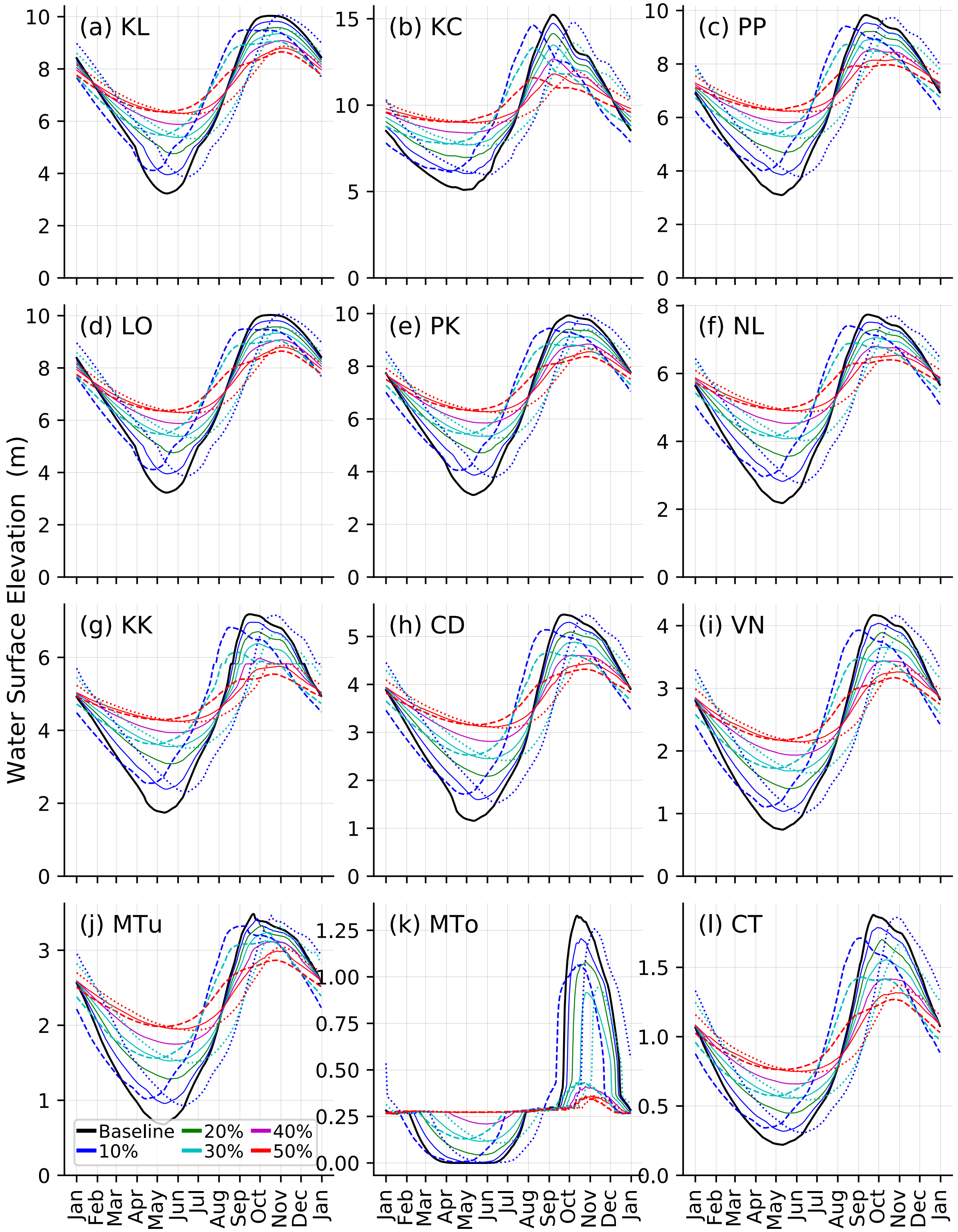

Figure S6. Same as in Fig. 4 in the main text but for simulated water level (i.e., water surface elevation).

6. Flooded Areas within the Entire Lower Mekong and Tonle Sap Lake Region

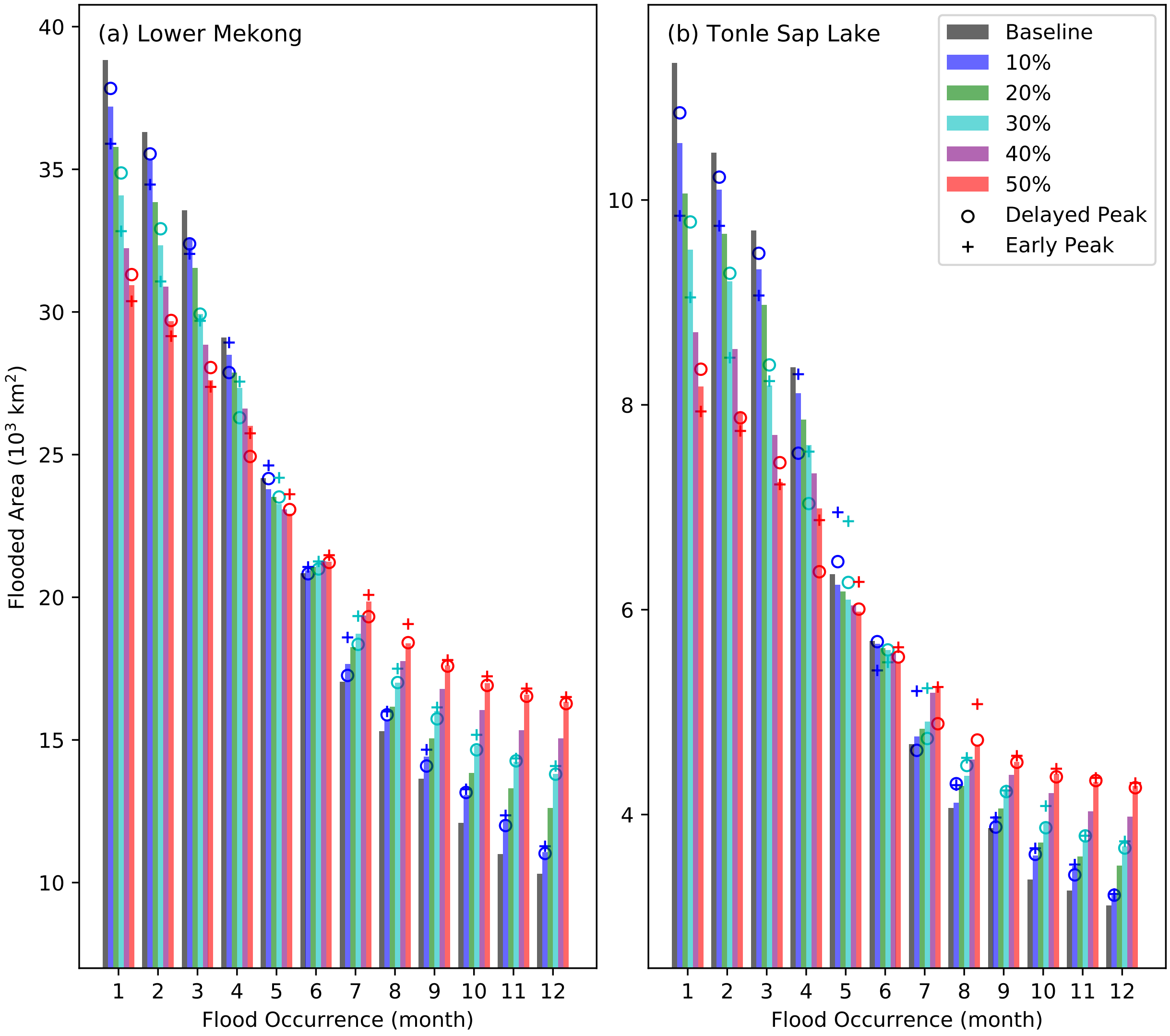

Figure S7. Flooded areas having different flood occurrence estimated from the baseline simulation results presented in Fig. 5a in the main text, and their changes under different flow regulation scenarios for (a) the entire domain shown in Figure 5a, and (b) the TSL region marked by thick black line within the red rectangle in Figure 5f in the main text. Plus signs and open circles show the results from the early and delayed peak flood timing by one month, respectively (see Methods in the main text).

7. Simulated Flood Occurrence during Dry and Wet Years.

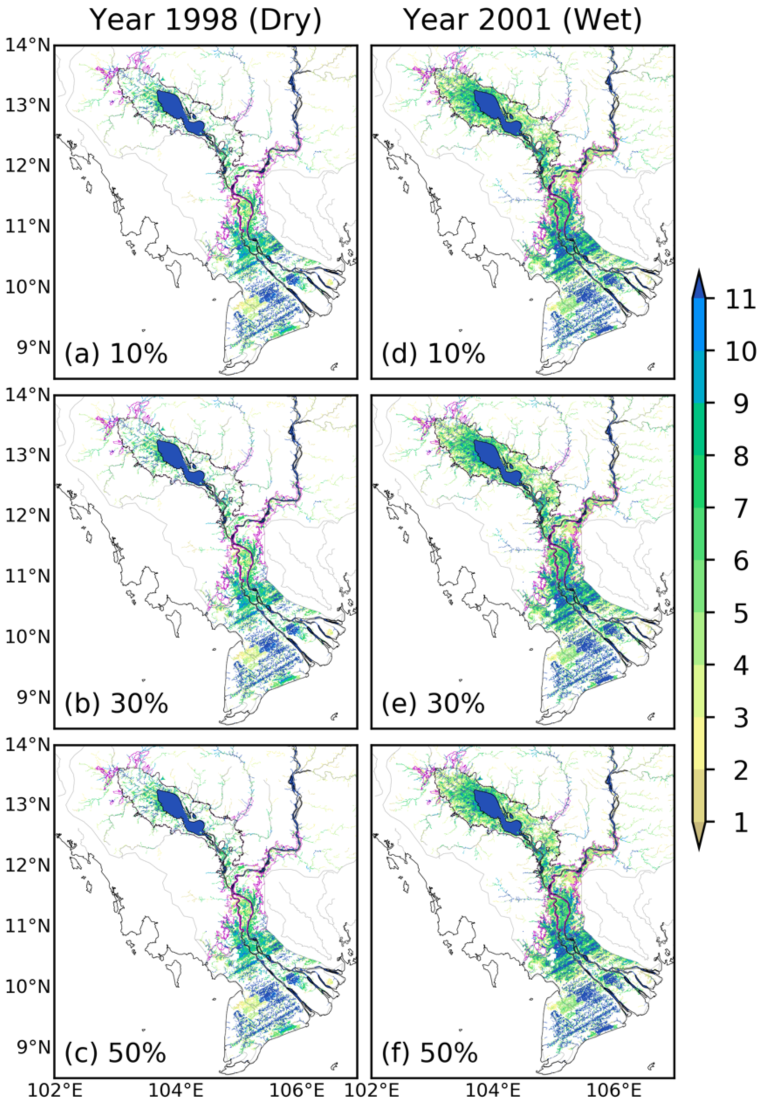

Figure S8. Simulated flood occurrence in dry and wet years for different flow regulation scenarios, i.e., the actual flood occurrence corresponding to the difference with the baseline shown in Figure 6b-d (1998) and 6f-h (2000).

8. Effects of Flow Regulation on Flood Dynamics in Dry (1998) and Wet (2000) Years

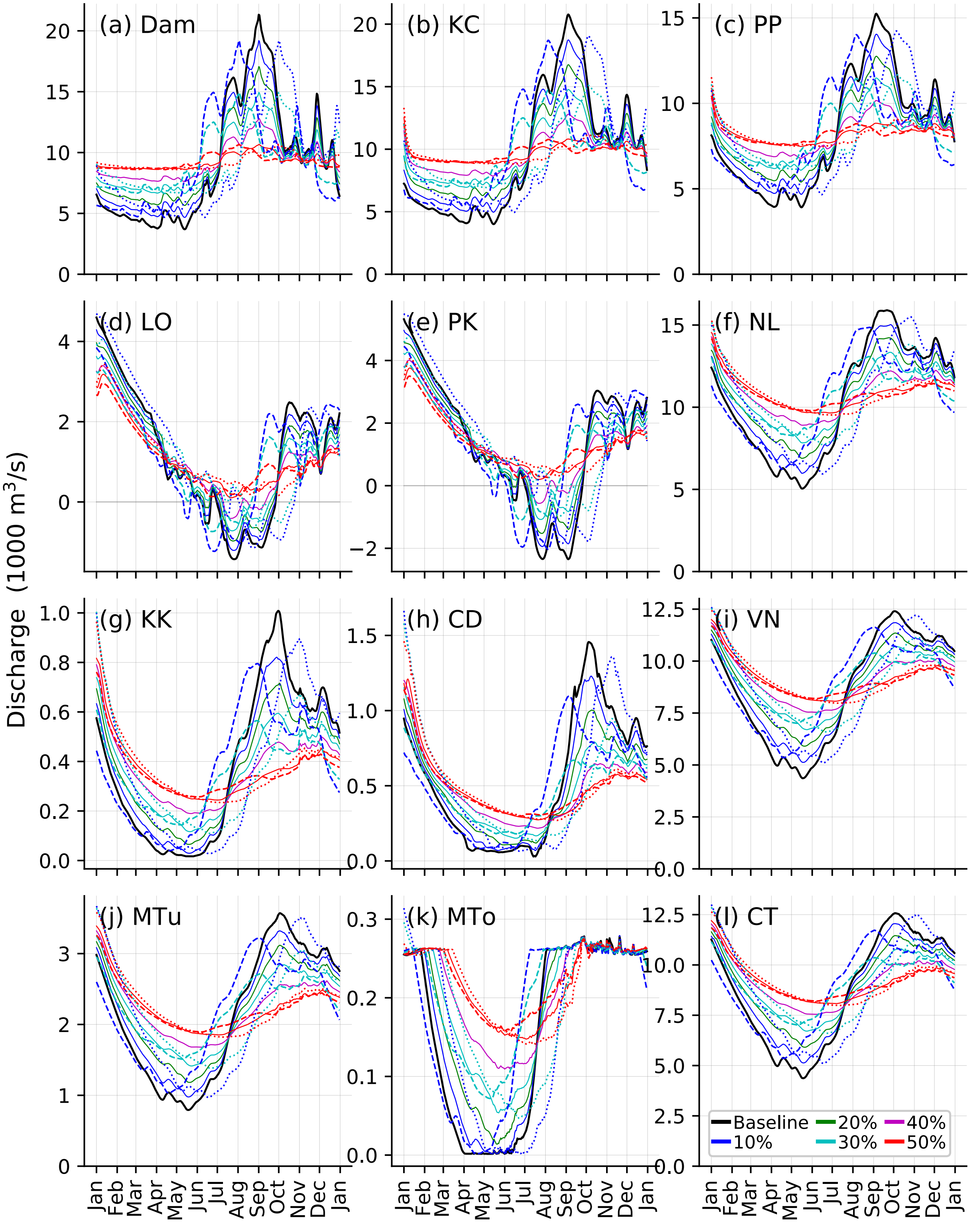

Figure S9. Same as in Fig. 4 in the main text but for 1998 (dry year).

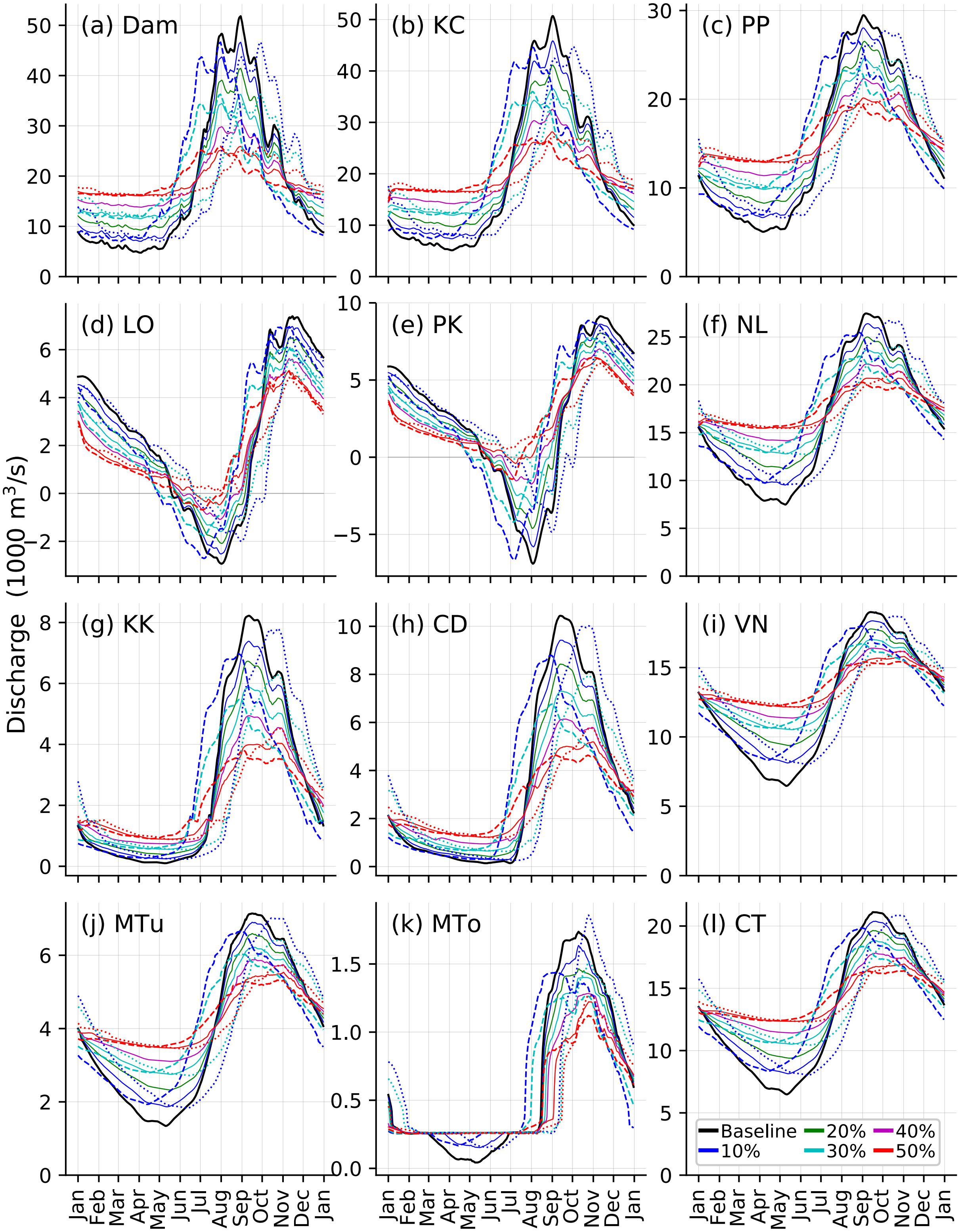

Figure S10. Same as in Fig. 4 in the main text but for 2000 (wet year).

9. Comparison of Flooded Areas with a Previous Study

Table S1. Comparison of flooded areas with Arias et al.[12] for the major flood regions around Tonle Sap Lake indicated by thick black line in Figure 1a in the main text.

Dry Season	Flooded Area (km^2)			Difference (%)[b]	
	MODIS[a]	GIS[a]	CaMa-Flood (this study)	MODIS	GIS
5/8/2000	2,841	3,072	3,442	17	11
4/15/2001	2,751	3,096	3,671	25	16
5/25/2002	2,580	2,433	3,144	18	23
6/2/2003	2,605	3,003	3,173	18	5
5/16/2004	2,579	2,281	3,100	17	26
5/1/2005	2,841	3,177	3,036	6	-5
5/1/2006	2,667	2,442	3,143	15	22
5/17/2007	2,626	3,029	3,405	23	11
**Wet Season**	Flooded Area (km^2)			Difference (%)	
	MODIS	GIS	CaMa-Flood (this study)	MODIS	GIS
10/23/2000	14,763	14,030	14,521	-2	3
10/8/2001	14,392	13,792	13,038	-10	-6
10/16/2002	14,264	13,103	12,517	-14	-5
10/24/2003	12,037	10,863	9,330	-29	-16
10/23/2004	12,264	10,894	10,669	-15	-2
10/16/2005	13,026	12,665	10,480	-24	-21
10/24/2006	13,180	12,624	13,635	3	7
10/16/2007	12,404	12,300	10,668	-16	-15

[a] MODIS and GIS data are from Arias et al.[12]

[b] $\text{Difference (\%)} = \frac{\text{CaMa-Flood} - \text{MODIS (or GIS)}}{\text{CaMa-Flood}} \times 100$

10. Changes in Flood Characteristics under Different Flow Regulation Scenarios

Table S2. Changes in major flood characteristics (e.g., onset, magnitude, duration, and amount) compared to the baseline simulation at different stations analyzed in Fig. 4 in the main text (station names provided in Fig. 4 caption and locations shown by red circles in Fig. 5a). The Lake Outlet (LO) and Prek Kdam (PK) stations are indicated by grey shading. Numbers enclosed in boxes are those noted in the main text. For 10, 30, and 50% peak flow alteration scenarios, results for the scenarios with one-month early and delayed peak timing are also provided.

(a) Q_{max} in baseline (m^3/s) and ΔQ_{max} (%)

Station	Baseline (m^3/sec)	10%	10% Delay	10% Early	20%	30%	30% Delay	30% Early	40%	50%	50% Delay	50% Early
Dam	36,329	-10	-10	-10	-20	-30	-30	-30	-40	-50	-50	-50
KC	35,747	-8	-8	-10	-17	-27	-26	-29	-37	-46	-46	-48
PP	22,450	-6	-5	-7	-12	-19	-17	-20	-27	-36	-34	-38
LO	4,479	-7	-16	-4	-14	-22	-29	-19	-29	-37	-42	-36
PK	5,423	-7	-17	-1	-13	-20	-27	-16	-27	-34	-37	-31
NL	21,105	-5	-2	-6	-9	-13	-10	-15	-17	-23	-20	-25
KK	4,049	-14	-2	-22	-29	-40	-30	-50	-58	-65	-62	-71
CD	4,931	-13	-1	-23	-28	-35	-29	-46	-52	-61	-56	-67
VN	15,578	-2	0	-4	-5	-9	-6	-12	-13	-16	-14	-19
MTu	5,408	-4	0	-11	-10	-16	-11	-23	-24	-28	-26	-32
MTo	1,028	-10	-5	-22	-20	-32	-30	-65	-67	-70	-70	-71
CT	16,704	-3	-1	-6	-7	-12	-8	-16	-17	-20	-18	-22

(b) Q_{min} in baseline (m^3/s) and ΔQ_{min} (m^3/s)

Station	Baseline (m^3/sec)	10%	10% Delay	10% Early	20%	30%	30% Delay	30% Early	40%	50%	50% Delay	50% Early
Dam	4,318	1,280	1,280	1,280	2,646	3,927	3,927	3,927	5,293	6,573	6,573	6,573
KC	4,645	1,264	1,235	1,311	2,625	3,907	3,875	3,967	5,287	6,547	6,528	6,587
PP	4,434	980	948	1,026	1,972	2,879	2,858	2,933	3,839	4,721	4,694	4,764
LO	-2,210	240 (-11%)	458 (-21%)	146 (-7%)	551 (-25%)	887 (-40%)	1,105 (-50%)	798 (-36%)	1,293 (-58%)	1,762 (-80%)	1,967 (-89%)	1,682 (-76%)
PK	-5,085	742 (-15%)	1,394 (-27%)	444 (-9%)	1,508 (-30%)	2,414 (-47%)	3,076 (-60%)	2,076 (-41%)	3,376 (-66%)	4,483 (-88%)	5,002 (-98%)	4,120 (-81%)
NL	5,749	1,244	1,131	1,509	2,709	3,865	3,754	4,062	4,965	5,921	5,843	6,038
KK	25	46	40	60	134	221	210	237	313	403	394	416
CD	69	36	31	45	123	210	203	223	300	396	387	415
VN	4,938	1,046	933	1,288	2,266	3,168	3,065	3,305	3,976	4,654	4,600	4,749
MTu	930	275	244	342	641	933	899	980	1,219	1,472	1,453	1,507
MTo	2	16	12	26	85	149	142	160	216	253	253	252
CT	4,948	1,049	934	1,288	2,268	3,170	3,067	3,307	3,968	4,638	4,587	4,727

(c) Q_{max} - Q_{min} in baseline (m^3/s) and $\Delta(Q_{max} - Q_{min})$ (%)

Station	Baseline (m^3/sec)	10%	10% Delay	10% Early	20%	30%	30% Delay	30% Early	40%	50%	50% Delay	50% Early
Dam	32,012	-15	-15	-15	-31	-46	-46	-46	-62	-77	-77	-77
KC	31,102	-13	-13	-15	-28	-43	-42	-46	-59	-74	-73	-76
PP	18,016	-12	-12	-14	-25	-39	-38	-41	-55	-71	-68	-73
LO	6,690	-8	-17	-5	-18	-28	-36	-25	-39	-51	-57	-49
PK	10,508	-10	-22	-5	-21	-33	-43	-28	-46	-60	-67	-55
NL	15,356	-15	-10	-18	-30	-43	-38	-47	-56	-70	-66	-73
KK	4,024	-15	-3	-23	-32	-46	-36	-57	-66	-75	-72	-82
CD	4,862	-14	-1	-24	-31	-40	-34	-52	-59	-70	-65	-77
VN	10,641	-13	-9	-18	-28	-43	-37	-48	-56	-68	-64	-72
MTu	4,478	-11	-6	-20	-26	-41	-33	-50	-57	-67	-64	-72
MTo	1,026	-12	-7	-24	-28	-47	-44	-81	-88	-94	-95	-95
CT	11,757	-13	-9	-19	-29	-44	-37	-51	-57	-69	-65	-72

(d) Onset of reversed flow in baseline (day of year) and its change (day)

Station	Baseline (DOY)	10%	10% Delay	10% Early	20%	30%	30% Delay	30% Early	40%	50%	50% Delay	50% Early
LO	159	1	32	-29	6	8	37	-20	16	38	74	7
PK	160	1	32	-29	7	9	42	-18	21	40	83	8

(e) Duration of reversed flow in baseline (day) and its change (day)

Station	Baseline (Days)	10%	10% Delay	10% Early	20%	30%	30% Delay	30% Early	40%	50%	50% Delay	50% Early
LO	112	-2	-7	0	-9	-13	-16	-13	-24	-51	-63	-46
PK	109	-2	-13	1	-10	-15	-23	-14	-31	-55	-100	-48

(f) Volume of reversed flow in baseline (10^6 m^3) and its change (%)

Station	Baseline (Mm^3)	10%	10% Delay	10% Early	20%	30%	30% Delay	30% Early	40%	50%	50% Delay	50% Early
LO	12,202	-14	-25	-9	-29	-47	-56	-43	-68	-87	-94	-84
PK	23,646	-15	-30	-7	-31	-50	-64	-43	-72	-92	-100	-86

Table S3. A summary of the results of potential changes in flooded area around Tonle Sap Lake under different flow regulation scenarios from this study and those from Arias et al.[13]. Flooded days with the smallest change are marked by grey shading. As noted in the main text, the results are not directly comparable due to differences in simulation settings between the two studies.

Flood occurrence[c] (%)	Flooded area (km^2) and its change from baseline simulations in km^2 (% in parentheses)[a]														
	Arias et al.[13]			This study											
	Baseline	DF[b]	DF+3S[b]	Baseline	10%	10% Delay	10% Early	20%	30%	30% Delay	30% Early	40%	50%	50% Delay	50% Early
≤ 10	16670	-94 (-0.6)	-120 (-0.7)	11129	-729 (-4.4)	74 (0.4)	-1404 (-8.4)	-1206 (-7.2)	-1627 (-9.8)	-1258 (-7.5)	-2180 (-13.1)	-2117 (-12.7)	-2891 (-17.3)	-2770 (-16.6)	-3147 (-18.9)
≤ 20	14912	-271 (-1.8)	-404 (-2.7)	10637	-433 (-2.9)	-249 (-1.7)	-1048 (-7.0)	-888 (-6.0)	-1434 (-9.6)	-1230 (-8.2)	-2191 (-14.7)	-2229 (-14.9)	-2759 (18.5)	-2669 (-17.9)	-2987 (-20.0)
≤ 30	13495	-322 (-2.4)	-569 (-4.2)	9729	-382 (-2.8)	-538 (-4.0)	-477 (-3.5)	-784 (-5.8)	-1408 (-10.4)	-1538 (-11.4)	-1543 (-11.4)	-1888 (-14.0)	-2411 (-17.9)	-2451 (-18.2)	-2563 (-19.0)
≤ 40	12074	-262 (-2.2)	-423 (-3.5)	7905	-208 (-1.7)	-352 (-2.9)	-12 (-0.1)	-448 (-3.7)	-692 (-5.7)	-814 (6.7)	-478 (-4.0)	-918 (-7.6)	-1148 (-9.5)	-1566 (-13.0)	-1101 (-9.1)
≤ 50	10407	-149 (-1.4)	-236 (-2.3)	6187	-63 (-0.6)	-124 (-1.2)	149 (1.4)	-147 (-1.4)	-236 (-2.3)	-323 (-3.1)	-18 (-0.2)	-301 (-2.9)	-427 (-4.1)	-470 (-4.5)	-251 (-2.4)
≤ 60	8874	-36 (-0.4)	-52 (-0.6)	4827	53 (0.6)	6 (0.1)	361 (4.1)	292 (3.3)	325 (3.7)	163 (1.8)	468 (5.3)	376 (4.2)	421 (-4.7)	399 (4.5)	527 (5.9)
≤ 70	7483	126 (1.7)	321 (4.3)	4186	74 (1.0)	91 (1.2)	171 (2.3)	171 (2.3)	270 (3.6)	261 (3.5)	365 (4.9)	383 (5.1)	515 (6.9)	491 (6.6)	619 (8.3)
≤ 80	6552	191 (2.9)	290 (4.4)	3631	125 (1.9)	143 (2.2)	186 (2.8)	248 (3.8)	506 (7.7)	456 (7.0)	584 (8.9)	685 (10.5)	839 (12.8)	833 (12.7)	895 (13.7)
≤ 90	5603	199 (3.6)	354 (6.3)	3244	57 (1.0)	69 (1.2)	242 (4.3)	410 (7.3)	569 (10.1)	543 (9.7)	634 (11.3)	911 (16.3)	1097 (19.6)	1096 (19.6)	1151 (20.5)
≤ 100	4910	278 (5.7)	424 (8.6)	3103	73 (1.5)	61 (1.2)	67 (1.4)	240 (4.9)	587 (11.9)	575 (11.7)	619 (12.6)	843 (17.2)	1171 (23.9)	1153 (23.5)	1211 (24.7)

[a] Spatial domain of Tonle Sap Lake region is exactly same as in Arias et al.[13].

[b] DF: Water infrastructure development plan up to 2015; DF+3S: Cumulative impact of the DF 42 dams in the main tributaries and 3S rivers (see Arias et al.[13] for details).

[c] To calculate flood occurrence, Arias et al.[13] used 15-years of simulations for 1986-2000 period, but this study uses 1-year of simulations driven by the climatological mean daily runoff for 1981-2010 period.

Table S4. Same as Table S2 but for dry year (1998). Blank fields in (d) and (e) denote no flow reversal.

(a) Q_{max} in baseline (m^3/s) and ΔQ_{max} (%)

Station	Baseline (m^3/sec)	10%	10% Delay	10% Early	20%	30%	30% Delay	30% Early	40%	50%	50% Delay	50% Early
Dam	21,327	-10	-10	-10	-20	-30	-30	-30	-40	-50	-50	-50
KC	20,775	-10	-8	-10	-19	-29	-27	-29	-38	-40	-36	-42
PP	15,249	-8	-7	-8	-16	-25	-23	-25	-33	-29	-24	-31
LO	4,597	-7	2	-16	-14	-21	-12	-28	-26	-31	-25	-36
PK	5,318	-7	3	-16	-13	-20	-10	-27	-25	-29	-24	-34
NL	15,904	-6	-3	-7	-11	-13	-5	-18	-10	-9	-4	-11
KK	1,008	-19	-1	-21	-29	-27	0	-40	-22	-19	-4	-24
CD	1,457	-16	14	-25	-26	-21	8	-39	-18	-17	0	-22
VN	12,402	-4	2	-6	-7	-6	1	-12	-4	-3	0	-6
MTu	3,571	-7	3	-10	-11	-9	2	-17	-7	-5	0	-9
MTo	278	0	13	-1	0	0	6	0	0	0	-1	0
CT	12,575	-4	3	-7	-7	-6	2	-11	-4	-3	1	-6

(b) Q_{min} in baseline (m^3/s) and ΔQ_{min} (m^3/s)

Station	Baseline (m^3/sec)	10%	10% Delay	10% Early	20%	30%	30% Delay	30% Early	40%	50%	50% Delay	50% Early
Dam	3,694	980	980	980	1,961	2,941	2,941	2,941	3,921	4,901	4,901	4,901
KC	4,005	955	933	996	1,916	2,886	2,860	2,921	3,848	4,826	4,800	4,876
PP	3,915	764	741	771	1,478	2,173	2,163	2,212	2,864	3,559	3,536	3,618
LO	-1,429	220<br>(-15%)	286<br>(-20%)	197<br>(-14%)	455<br>(-32%)	708<br>(-50%)	777<br>(-54%)	691<br>(-48%)	1,017<br>(-71%)	1,529<br>(-107%)	1,591<br>(-111%)	1,560<br>(-109%)
PK	-2,351	407<br>(-17%)	506<br>(-22%)	317<br>(-14%)	827<br>(-35%)	1,270<br>(-54%)	1,384<br>(-59%)	1,257<br>(-53%)	1,785<br>(-76%)	2,552<br>(-109%)	2,663<br>(-113%)	2,598<br>(-111%)
NL	5,045	933	898	1,184	1,840	2,785	2,677	3,219	3,858	4,601	4,479	4,743
KK	16	13	12	24	49	101	93	128	174	228	218	240
CD	29	40	36	53	78	122	106	150	189	244	241	269
VN	4,359	764	735	990	1,544	2,339	2,233	2,649	3,176	3,707	3,615	3,829
MTu	792	185	178	242	390	622	591	719	887	1,061	1,029	1,102
MTo	2	0	0	0	12	52	45	70	108	146	140	156
CT	4,367	762	734	988	1,541	2,342	2,232	2,647	3,179	3,706	3,615	3,833

(c) Q_{max} - Q_{min} in baseline (m^3/s) and $\Delta(Q_{max} - Q_{min})$ (%)

Station	Baseline (m^3/sec)	10%	10% Delay	10% Early	20%	30%	30% Delay	30% Early	40%	50%	50% Delay	50% Early
Dam	17,634	-18	-18	-18	-35	-53	-53	-53	-71	-88	-88	-88
KC	16,770	-18	-16	-18	-35	-53	-51	-53	-71	-78	-73	-81
PP	11,334	-17	-15	-18	-35	-52	-50	-53	-69	-70	-64	-74
LO	6,026	-9	-3	-16	-18	-28	-22	-33	-37	-49	-46	-53
PK	7,669	-10	-4	-15	-20	-30	-25	-35	-40	-54	-51	-58
NL	10,858	-17	-12	-20	-32	-45	-32	-56	-51	-55	-47	-60
KK	992	-20	-2	-24	-34	-37	-10	-53	-39	-42	-26	-49
CD	1,428	-19	12	-29	-32	-30	1	-51	-31	-34	-17	-41
VN	8,043	-16	-7	-22	-30	-38	-26	-51	-46	-51	-44	-56
MTu	2,779	-16	-3	-21	-29	-34	-19	-48	-41	-45	-37	-51
MTo	277	0	13	-1	-5	-19	-10	-26	-39	-53	-51	-57
CT	8,208	-16	-4	-22	-30	-37	-24	-50	-45	-50	-43	-56

(d) Onset of reversed flow in baseline (day of year) and its change (day)

Station	Baseline (DOY)	10%	10% Delay	10% Early	20%	30%	30% Delay	30% Early	40%	50%	50% Delay	50% Early
LO	159	2	32	-27	4	30	62	0	32	-	-	-
PK	162	0	30	-30	2	28	59	-1	30	-	-	-

(e) Duration of reversed flow in baseline (day) and its change (day)

Station	Baseline (Days)	10%	10% Delay	10% Early	20%	30%	30% Delay	30% Early	40%	50%	50% Delay	50% Early
LO	103	-6	-9	-7	-15	-31	-32	-29	-48	-	-	-
PK	96	-5	-8	-3	-13	-27	-33	-25	-45	-	-	-

(f) Volume of reversed flow in baseline (10^6 m^3) and its change (%)

Station	Baseline (Mm^3)	10%	10% Delay	10% Early	20%	30%	30% Delay	30% Early	40%	50%	50% Delay	50% Early
LO	7,245	-22	-27	-16	-43	-65	-69	-60	-86	-100	-100	-100
PK	12,180	-23	-30	-18	-46	-68	-74	-63	-89	-100	-100	-100

Table S5. Same as Table S2 but for wet year (2000). Blank fields in (d) and (e) denote no flow reversal.

(a) Q_{max} in baseline (m^3/s) and ΔQ_{max} (%)

Station	Baseline (m^3/sec)	10%	10% Delay	10% Early	20%	30%	30% Delay	30% Early	40%	50%	50% Delay	50% Early
Dam	51,826	-10	-10	-10	-20	-30	-30	-30	-40	-50	-50	-50
KC	50,684	-10	-12	-12	-19	-28	-29	-29	-36	-44	-46	-47
PP	29,477	-5	-6	-7	-10	-17	-17	-20	-24	-32	-30	-34
LO	7,396	-6	-11	-6	-12	-18	-24	-17	-24	-31	-35	-31
PK	9,135	-6	-10	-3	-12	-18	-24	-15	-23	-30	-33	-29
NL	27,471	-4	-3	-7	-9	-14	-12	-16	-19	-25	-22	-26
KK	8,222	-10	-5	-15	-18	-28	-24	-33	-40	-51	-45	-54
CD	10,441	-10	-4	-16	-19	-30	-24	-35	-41	-50	-45	-56
VN	18,999	-3	-1	-5	-6	-10	-8	-12	-14	-17	-15	-19
MTu	7,140	-3	-2	-7	-8	-13	-10	-16	-18	-22	-20	-25
MTo	1,737	-6	7	-17	-16	-19	-13	-27	-26	-30	-27	-35
CT	21,128	-3	-1	-6	-7	-11	-9	-13	-16	-20	-17	-22

(b) Q_{min} in baseline (m^3/s) and ΔQ_{min} (m^3/s)

Station	Baseline (m^3/sec)	10%	10% Delay	10% Early	20%	30%	30% Delay	30% Early	40%	50%	50% Delay	50% Early
Dam	4,733	2,266	2,266	2,266	4,532	6,798	6,798	6,798	9,065	11,331	11,331	11,331
KC	5,107	2,247	2,258	2,287	4,569	6,817	6,814	6,627	9,055	10,160	11,286	9,451
PP	5,042	1,610	1,555	1,776	3,233	4,794	4,760	4,898	6,338	7,835	7,806	7,313
LO	-2,935	410 (-14%)	931 (-30%)	226 (-4%)	850 (-29%)	1,349 (-46%)	1,787 (-61%)	1,174 (-40%)	1,852 (-63%)	2,461 (-84%)	3,007 (-102%)	2,247 (-77%)
PK	-6,902	1,090 (-16%)	2,052 (-30%)	248 (-4%)	2,294 (-33%)	3,701 (-54%)	4,510 (-65%)	2,702 (-39%)	5,167 (-75%)	6,497 (-94%)	7,444 (-108%)	5,481 (-79%)
NL	7,473	2,051	1,960	2,264	3,772	5,306	5,274	5,449	6,670	7,985	7,981	8,006
KK	99	143	135	163	291	457	450	475	634	788	782	802
CD	138	139	116	166	286	502	489	533	804	1,083	1,063	1,136
VN	6,466	1,652	1,546	1,862	2,914	4,047	3,980	4,153	4,889	5,685	5,668	5,756
MTu	1,342	522	487	590	982	1,406	1,379	1,452	1,763	2,123	2,113	2,155
MTo	41	111	102	125	213	213	213	214	214	213	213	213
CT	6,484	1,654	1,542	1,857	2,904	4,032	3,959	4,146	4,932	5,858	5,837	5,900

(c) Q_{max} - Q_{min} in baseline (m^3/s) and $\Delta(Q_{max} - Q_{min})$ (%)

Station	Baseline (m^3/sec)	10%	10% Delay	10% Early	20%	30%	30% Delay	30% Early	40%	50%	50% Delay	50% Early
Dam	47,094	-16	-16	-16	-32	-47	-47	-47	-63	-79	-79	-79
KC	45,577	-16	-18	-18	-31	-46	-48	-47	-60	-72	-76	-73
PP	24,436	-12	-14	-15	-25	-40	-40	-45	-55	-70	-68	-71
LO	10,332	-8	-17	-7	-17	-26	-35	-24	-35	-46	-54	-44
PK	16,037	-10	-19	-3	-21	-33	-42	-25	-45	-58	-66	-51
NL	19,998	-16	-14	-21	-31	-46	-44	-50	-60	-74	-70	-76
KK	8,123	-12	-7	-17	-22	-34	-29	-40	-48	-61	-55	-64
CD	10,303	-11	-5	-18	-22	-35	-29	-41	-50	-61	-56	-67
VN	12,532	-18	-15	-23	-33	-48	-44	-51	-60	-71	-68	-74
MTu	5,798	-13	-11	-19	-26	-40	-36	-44	-52	-64	-61	-69
MTo	1,696	-12	1	-25	-29	-32	-26	-41	-39	-43	-40	-49
CT	14,644	-16	-12	-21	-30	-43	-40	-47	-56	-68	-65	-72

(d) Onset of reversed flow in baseline (day of year) and its change (day)

Station	Baseline (DOY)	10%	10% Delay	10% Early	20%	30%	30% Delay	30% Early	40%	50%	50% Delay	50% Early
LO	141	6	32	-21	7	11	35	-17	14	34	-	6
PK	143	5	33	-21	7	10	41	-18	14	36	-	5

(e) Duration of reversed flow in baseline (day) and its change (day)

Station	Baseline (Days)	10%	10% Delay	10% Early	20%	30%	30% Delay	30% Early	40%	50%	50% Delay	50% Early
LO	114	-8	-7	-6	-12	-21	-32	-14	-28	-66	-	-45
PK	111	-7	-13	-7	-13	-23	-57	-14	-53	-80	-	-60

(f) Volume of reversed flow in baseline (10^6 m^3) and its change (%)

Station	Baseline (Mm^3)	10%	10% Delay	10% Early	20%	30%	30% Delay	30% Early	40%	50%	50% Delay	50% Early
LO	15,181	-18	-40	-7	-38	-57	-75	-46	-75	-92	-100	-86
PK	31,642	-20	-49	-3	-42	-63	-85	-46	-82	-99	-100	-89

Table S6. Geographic location of stations used in Figures 1, 4, S2, S3, S4, S5, S8, and S9.

Station	Name	Latitude	Longitude	Classification
LP	Luang Prabang	19.89	102.14	Mainstream Mekong
PA	Pakse	15.12	105.80	Mainstream Mekong
ST	Stung Treng	13.53	105.95	Mainstream Mekong
Dam	Dam	13.55	105.95	Mainstream Mekong
KT	Kratie	12.49	106.02	Mainstream Mekong
KC	Kampong Cham	12.00	105.47	Mainstream Mekong
KL	Kompong Luong	12.58	104.22	Tonle Sap RIver
LO	Lake Outlet	12.52	104.47	Tonle Sap RIver
PK	Prek Kdam	11.81	104.80	Tonle Sap RIver
PP	Phnom Penh Port	11.58	104.92	Mainstream Mekong
NL	Neak Luong	11.26	105.28	Mainstream Mekong
KK	Koh Khel	11.27	105.02	Bassac River
CD	Chau Doc	10.70	105.13	Bassac River
VN	Vam Nao	10.58	105.36	Mainstream Mekong
MTu	My Thuan	10.27	105.92	Mekong Delta
MTo	My Tho	10.36	106.37	Mekong Delta
CT	Can Tho	10.03	105.79	Bassac River/Song Hau

References

1 Pokhrel, Y., Fan, Y., Miguez-Macho, G., Yeh, P. J. F. & Han, S.-C. The role of groundwater in the Amazon water cycle: 3. Influence on terrestrial water storage computations and comparison with GRACE. *J. Geophys. Res. Atmos.* **118**, 3233-3244, doi:10.1002/jgrd.50335 (2013).

2 Tapley, B. D., Bettadpur, S., Ries, J. C., Thompson, P. F. & Watkins, M. M. GRACE Measurements of Mass Variability in the Earth System. *Science* **305**, 503-505, doi:10.1126/science.1099192 (2004).

3 Pokhrel, Y. *et al.* Incorporation of groundwater pumping in a global Land Surface Model with the representation of human impacts. *Water Resources Research* **51**, 78-96, doi:10.1002/2014WR015602 (2015).

4 Landerer, F. W. & Swenson, S. C. Accuracy of scaled GRACE terrestrial water storage estimates. *Water Resources Research* **48**, W04531, doi:10.1029/2011WR011453 (2012).

5 Scanlon, B. R. *et al.* Global evaluation of new GRACE mascon products for hydrologic applications. *Water Resources Research* **52**, 9412-9429 (2016).

6 Felfelani, F., Wada, Y., Longuevergne, L. & Pokhrel, Y. Natural and human-induced terrestrial water storage change: A global analysis using hydrological models and GRACE. *Journal of Hydrology* **553**, 105-118 (2017).

7 Syed, T. H., Famiglietti, J. S. & Chambers, D. P. GRACE-Based Estimates of Terrestrial Freshwater Discharge from Basin to Continental Scales. *Journal of Hydrometeorology* **10**, 22-40, doi:10.1175/2008jhm993.1 (2009).

8 Yeh, P. J. F., Swenson, S. C., Famiglietti, J. S. & Rodell, M. Remote sensing of groundwater storage changes in Illinois using the Gravity Recovery and Climate Experiment (GRACE). *Water Resources Research* **42**, W12203, doi:10.1029/2006WR005374 (2006).

9 Oki, T. & Sud, Y. C. Design of Total Runoff Integrating Pathways (TRIP)—A Global River Channel Network. *Earth Interact.* **2**, 1-37, doi:10.1175/1087-3562(1998)002<0001:DOTRIP>2.3.CO;2 (1998).

10 Yamazaki, D., Sato, T., Kanae, S., Hirabayashi, Y. & Bates, P. D. Regional flood dynamics in a bifurcating mega delta simulated in a global river model. *Geophysical Research Letters* **41**, 3127-3135 (2014).

11 Farr, T. G. *et al.* The shuttle radar topography mission. *Rev. Geophys.* **45** (2007).

12 Arias, M. *et al.* Quantifying changes in flooding and habitats in the Tonle Sap Lake (Cambodia) caused by water infrastructure development and climate change in the Mekong Basin. *Journal of Environmental Management* **112**, 53-66, doi:10.1016/j.jenvman.2012.07.003 (2012).

13 Arias, M., Piman, T., Lauri, H., Cochrane, T. & Kummu, M. Dams on Mekong tributaries as significant contributors of hydrological alterations to the Tonle Sap Floodplain in Cambodia. *Hydrology and Earth System Sciences* **18**, 5303 (2014).
